# Robust and accurate Bayesian inference of genome-wide genealogies for hundreds of genomes

**DOI:** 10.1038/s41588-025-02317-9

**Published:** 2025-09-08

**Authors:** Yun Deng, Rasmus Nielsen, Yun S. Song

**Affiliations:** 1 Center for Computational Biology, University of California, Berkeley, CA USA; 2 Department of Statistics, University of California, Berkeley, CA USA; 3 Department of Integrative Biology, University of California, Berkeley, CA USA; 4 Center for GeoGenetics, University of Copenhagen, Copenhagen, Denmark; 5 Computer Science Division, University of California, Berkeley, CA USA

**Keywords:** Population genetics, Software

## Abstract

The Ancestral Recombination Graph (ARG), which describes the genealogical history of a sample of genomes, is a vital tool in population genomics and biomedical research. Recent advancements have substantially increased ARG reconstruction scalability, but they rely on approximations that can reduce accuracy, especially under model misspecification. Moreover, they reconstruct only a single ARG topology and cannot quantify the considerable uncertainty associated with ARG inferences. Here, to address these challenges, we introduce SINGER (sampling and inferring of genealogies with recombination), a method that accelerates ARG sampling from the posterior distribution by two orders of magnitude, enabling accurate inference and uncertainty quantification for hundreds of whole-genome sequences. Through extensive simulations, we demonstrate SINGER’s enhanced accuracy and robustness to model misspecification compared to existing methods. We demonstrate the utility of SINGER by applying it to individuals of British and African descent within the 1000 Genomes Project, identifying signals of population differentiation, archaic introgression and strong support for ancient polymorphism in the human leukocyte antigen region shared across primates.

## Abstract (web summary)

SINGER is a method for creating ancestral recombination graphs to understand the genealogical history of genomes. The method has increased speed, and thus scalability, without sacrificing accuracy.

## Main

Many problems in genomics rely on computationally inferring genealogical relationships from large collections of DNA sequences and interpreting the reconstructed trees. In particular, genealogical approaches have been instrumental in understanding human genetic variation^1–3^ and underpin numerous computational methods used in biomedical research. In species with recombination, such as humans, the genealogical history cannot be represented by a single tree. Instead, each genomic position has its own tree that minimally differs from those at neighboring positions, resulting in millions of trees across the genome. The collection of these trees, along with recombination points, is represented by the ARG, and the associated generative model is known as ‘the coalescent with recombination’^4,5^.

Although simulating under the coalescent with recombination is straightforward^6–8^, inferring ARGs from genetic variation data remains a major challenge owing to the enormous space of possible ARGs. ARGs can be built iteratively by determining where the *n*^th^ lineage joins the partial ARG for the first *n* − 1 genomes, a process referred to as ‘threading’. By using an approximation known as the sequentially Markov coalescent (SMC)^9–11^ and formulating the threading problem as a hidden Markov model (HMM)^12–14^, in combination with a clever Markov chain Monte Carlo (MCMC) method, ARGweaver^15^ can sample genome-wide ARGs from the approximate posterior distribution for tens of whole-genome sequences. However, it is computationally intensive, rendering it impractical for larger sample sizes.

Recently, significant advances have been made in scaling up ARG reconstruction to tens or hundreds of thousands of genomes. Relate^16^ and tsinfer + tsdate^1,3^ use an efficient HMM (namely the Li–Stephens model)^17^ to infer local tree topologies along the genome, followed by branch length estimation. ARG-Needle^18^ uses a threading approach similar to ARGweaver but incorporates several heuristics to enhance scalability. These improvements have enabled new research directions^19–23^ and have facilitated diverse ARG-based applications in population and statistical genetics^24–35^.

Despite the progress, there are substantial limitations in current ARG inference methods that impede ARG-based analyses of whole-genome sequencing (WGS) data. First, improved scalability usually comes at the cost of accuracy in key ARG features, such as coalescence times^36^ and recombination events^37^. For instance, Relate and tsinfer + tsdate perform sub-optimally for ancient coalescence times, diminishing their effectiveness in applications involving ancient times, such as detecting balancing selection. Second, most scalable methods reconstruct only a single ARG topology and overlook estimation uncertainty. As we demonstrate in this article, accurate sampling of ARGs improves statistical inference, especially for local gene tree analysis, in which point estimates are often noisy. Applications such as local ancestry inference, introgression detection and selection analysis would be challenging without proper confidence intervals. For instance, CLUES^34^, a method for inferring selection and allele frequency trajectories, performs better with a sample of local trees. Third, current methods typically assume simple priors, such as a constant-size panmictic population and neutrality, and are not robust against violations of these assumptions. This limitation has consequential implications for applications, as many human populations have undergone complex demographic changes, including bottlenecks and recent expansions^12,38^. Similarly, the influence of background selection is pervasive, profoundly shaping the diversity landscape^39,40^.

To address these challenges, we introduce SINGER, a Bayesian method for ARG inference. SINGER retains all functionalities of ARGweaver—including MCMC-based posterior sampling, topology exploration, tracking of recombination events and so on—while being at least an order of magnitude faster. Through extensive simulations, we demonstrate that SINGER attains higher accuracy than competing methods in several crucial aspects of ARG inference. It also exhibits greater robustness to various model misspecifications. We highlight the utility of our method by applying it to British and African individuals within the 1000 Genomes Project, revealing signals of population differentiation in coalescence times and archaic introgression and providing strong evidence of trans-species polymorphism and balancing selection in human leukocyte antigen (HLA) regions.

## Results

### An overview of the SINGER algorithm

**Fig. 1:**
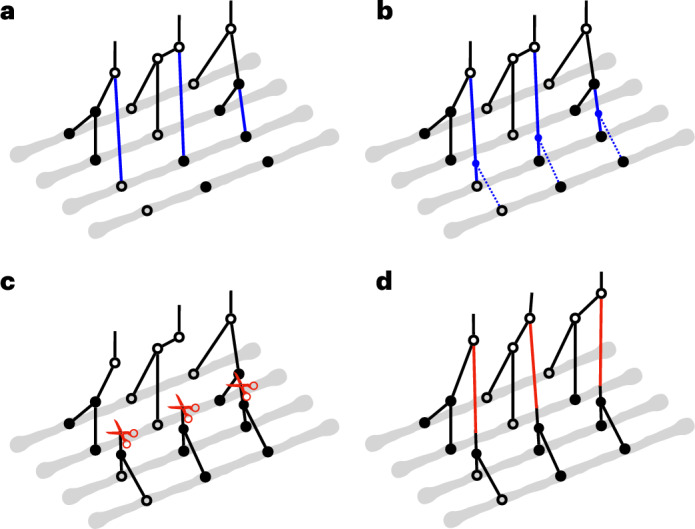
Method overview. **a**–**d**, The gray lines represent haplotypes, and the circles indicate the allelic states of nodes in coalescent trees. Hollow circles correspond to ancestral alleles, and solid circles are derived alleles. In **a**, **b** and **c**, a partial ARG for the first three haplotypes has already been constructed, and a fourth haplotype is about to be threaded onto this partial ARG. **a**, The initial step in threading the fourth haplotype involves sampling the joining branch (highlighted in blue) in each marginal coalescent tree of the partial ARG, a process we call ‘branch sampling’. **b**, Following the determination of the joining branches, the next step is to sample the joining time for each of these joining branches. This step is referred to as ‘time sampling’. **c**,**d**, To propose an update to an ARG in MCMC, we first introduce cuts (illustrated by red scissors) to a sequence of marginal coalescent trees to prune subtrees. Then we re-graft them by solving the threading problem for the sub-ARG above the cuts. The branch length in the first two marginal trees and the topology of the third tree are altered after ‘sub-graph pruning and re-grafting’.

SINGER takes in phased WGS data and samples ARGs iteratively by adding one haplotype at a time through an operation called threading^15^. Conditioned on a partial ARG for the first *n* − 1 haplotypes, the threading operation samples the points at which the lineage for the *n*^th^ haplotype joins the partial ARG. SINGER solves this by first building an HMM with branches as hidden states and sampling a sequence of joining branches along the genome from the posterior, using stochastic traceback (Fig. 1a). Then, SINGER builds another HMM with joining times as hidden states, conditioned on these sampled joining branches (Fig. 1b). We refer to these two steps as ‘branch sampling’ and ‘time sampling’, respectively (Methods). Although this two-step threading algorithm is approximative, by substantially reducing the number of hidden states, it is much faster than ARGweaver’s HMM, which treats every joining point in the tree as a hidden state.

To explore the space of ARG topology and branch lengths according to the posterior distribution, SINGER uses an MCMC proposal called ‘sub-graph pruning and re-grafting’ (SGPR). In brief, an SGPR operation first prunes a sub-graph by introducing a cut and then extends it leftwards and rightwards (Fig. 1c); in Supplementary Section B.4, we show that the pruning step is equivalent to the removal step in the so-called ‘Kuhner move’^41,42^. However, our re-graft step (Fig. 1d) differs substantially from the Kuhner move; the latter samples from the prior by simulation, whereas SGPR uses the threading algorithm to sample from the posterior. Given that the Kuhner move ignores data during re-grafting, it rarely improves likelihood, whereas SGPR favors data-compatible updates. Compared to the Kuhner move and ARGweaver, SGPR introduces large updates to the ARG with higher acceptance rates (Supplementary Section B.4 and Supplementary Fig. 1), yielding a better convergence rate and mixing of the MCMC.

Lastly, to mitigate biases introduced by algorithmic approximations, SINGER performs ‘ARG re-scaling’ through a monotonic transformation of node times that aligns the inferred mutation density with branch lengths (Methods). This is conceptually similar to the ‘ARG normalization’ procedure introduced in ARG-Needle^18^, but ARG-Needle uses a provided demographic prior, whereas SINGER learns the transformation from the inferred ARG without external information. As long as the relative ordering of node ages is accurate, ARG re-scaling can calibrate the overall time distribution. In simulation benchmarks, we show that this greatly improves robustness against model misspecification (for example, population size changes) even though the HMMs assume a constant population size. This approach parallels site frequency spectra-based demography methods^43–45^ but incorporates explicit tree topologies inferred from SINGER, offering greater robustness to changing population sizes.

### Performance benchmarks on simulated data

We first benchmarked the performance of several ARG inference methods (SINGER, ARGweaver, Relate, tsinfer + tsdate, ARG-Needle) using data simulated with msprime^46^. The simulation setup and benchmarking procedures are detailed in the Methods.

#### Coalescence time accuracy

**Fig. 2:**
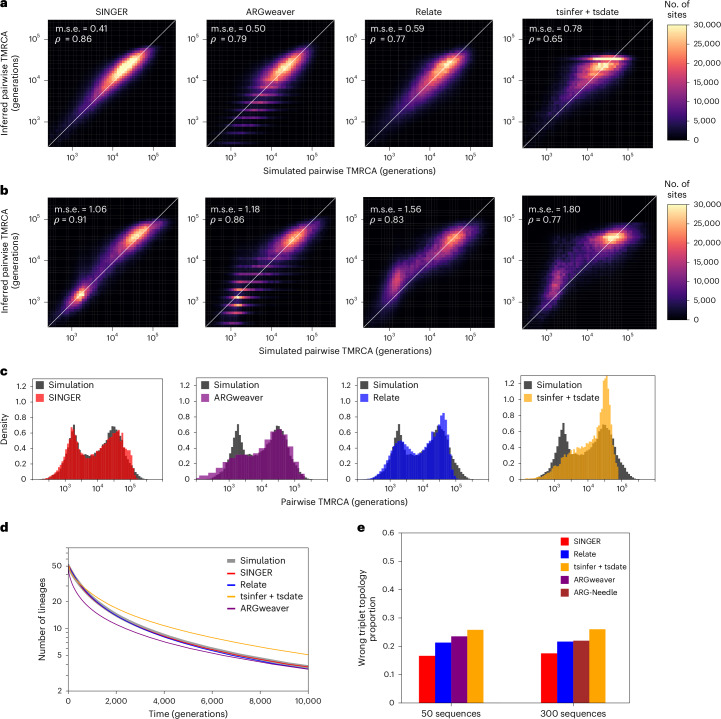
Performance benchmarks on coalescence time and topology inference. **a**, Inferred pairwise coalescence times compared with the ground truth in simulations involving 50 sequences under a constant population size scenario. **b**, Similar to **a**, but for data simulated under an inferred population size history for the CEU population. **c**, Inferred distribution of pairwise coalescence times (colored) compared with the ground truth (dark gray) from simulations under the same CEU demography as in **b**. **d**, Genome-wide average of the number of lineages as a function of time for 50 sequences under a constant population size history, compared with the ground truth in simulations. **e**, The proportion of triplet topologies that are incorrectly inferred for 50 and 300 sequences under a constant population size history. Owing to runtime constraints, ARGweaver is not benchmarked for 300 sequences.

**Extended Data Fig. 1:**
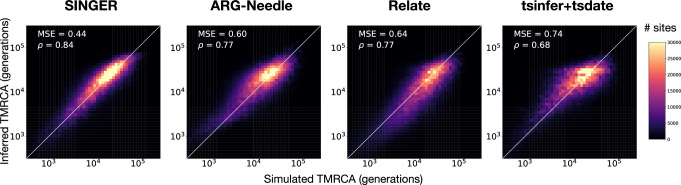
Inference of pairwise TMRCA on 300 sequences. Performance of inferring pairwise TMRCA with SINGER, ARG-Needle, Relate and tsinfer+tsdate, with 300 sequences. SINGER performs the best, while ARG-Needle and Relate perform similarly, and tsinfer+tsdate performs the worst.

To evaluate coalescence time estimation, we compared the ground truth and the inferred pairwise coalescence times for 100 randomly chosen leaf-node pairs, following a previous publication^36^. Pairwise coalescence times are important for applications such as demography inference^16^, genome-wide association studies^18,25,47^ and evolutionary studies^35^. For 50 haplotypes, SINGER was the most accurate; ARGweaver and Relate performed similarly, while tsinfer + tsdate was the least accurate (Fig. 2a). For 300 haplotypes, we compared only SINGER, Relate, tsinfer + tsdate and ARG-Needle, as this sample size is too large for ARGweaver. SINGER again performed best; Relate and ARG-Needle performed similarly and tsinfer + tsdate remained the least accurate (Extended Data Fig. 1). SINGER’s improved performance over ARGweaver might reflect better MCMC mixing efficiency and more flexible time discretization.

We also compared against pairwise-coalescent methods that analyze each sequence pair independently, specifically considering the recently proposed method Gamma-SMC^48^. SINGER substantially outperforms Gamma-SMC (Fig. 2a and Supplementary Fig. 2), whereas Relate and tsinfer + tsdate show no improvement in either mean squared error or correlation.

We also evaluated the genome-wide average number of lineages as a function of time in marginal trees, a statistic relevant to demography and selection inference. ARGweaver underestimates many recent coalescence times, causing the number of lineages to drop too fast (Fig. 2d and Supplementary Fig. 3); this aligns with Fig. 2a and corroborates a previous finding^16^ that ARGweaver tends to underestimate times. On the other hand, tsdate substantially overestimates coalescence times (Fig. 2d). By contrast, Relate and SINGER agree well with the expectation (Fig. 2d).

#### Tree topology accuracy

To assess topology inference accuracy, we used the triplet distance, defined as the fraction of three-leaved subtrees with different topologies in a given pair of trees. This metric is relevant for applications such as imputation and local ancestry, which depend on the accuracy of local topologies. On average, SINGER achieved the lowest triplet distances to the ground truth (Fig. 2e). Again, ARGweaver was less accurate than SINGER, potentially owing to ARGweaver’s less efficient MCMC and the presence of polytomies in its inferred trees. We also considered an evaluation metric related to the total variation distance introduced in a previous work^18^, and the results similarly favored SINGER over other methods (Supplementary Section C.2 and Supplementary Figs. 4 and 5).

#### Robustness to model misspecification

One advantage of SINGER is its robustness to model misspecification; specifically, it is less sensitive to incorrect effective population sizes $\,{(N}_{e})$ and unmodeled population size changes. When using an ${N}_{e}$ that is off by a factor of five, the coalescent times inferred by SINGER were less biased than Relate and tsinfer + tsdate, which showed systematic underestimation (Supplementary Fig. 6).

We simulated data under an inferred CEU population size history^49,50^, which contains a bottleneck and recent expansion. On these data, SINGER not only inferred the coalescence times more accurately than ARGweaver, Relate and tsinfer + tsdate (Fig. 2b) but also accurately captured the bi-modality in the pairwise coalescence time distribution caused by the bottleneck (Fig. 2c). Although Relate can incorporate population size changes, it requires running a separate module of estimating branch lengths and coalescent rates, which takes even longer than running Relate itself. ARG-Needle requires a user-specified size history to adjust its coalescence times and is not able to handle an unknown size history. By contrast, SINGER automatically adjusts branch lengths through ARG re-scaling, with little computational overhead.

#### Accuracy of mutation and recombination inferences

**Fig. 3:**
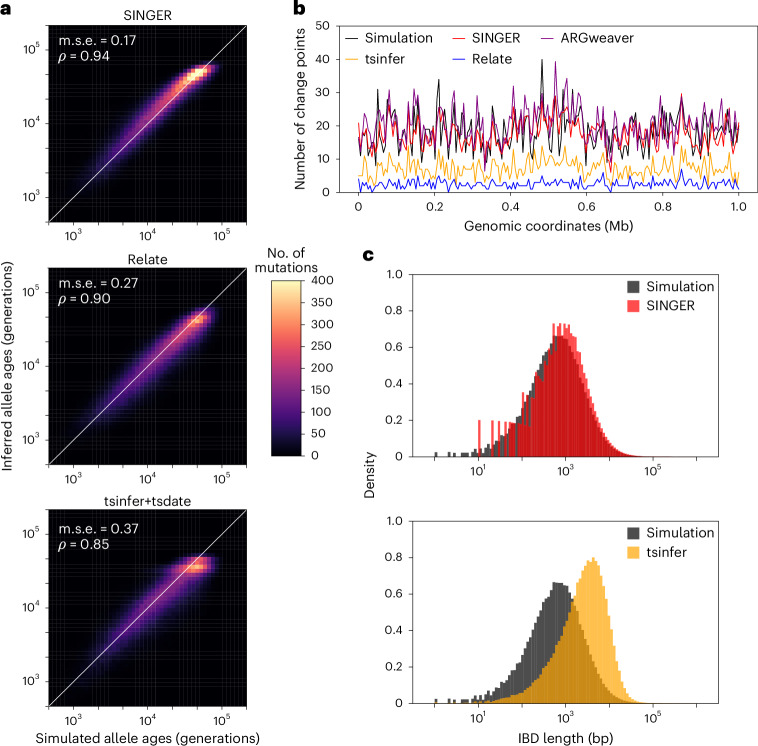
Benchmarks on mutation and recombination inference for data simulated with 50 sequences and constant population size. **a**, Inferred allele ages compared with the ground truth. **b**, Inferred number of recombination breakpoints in 5 kb genomic windows compared with the ground truth. **c**, The length distribution of pairwise IBD in the inferred ARGs compared with the ground truth.

**Extended Data Fig. 2:**
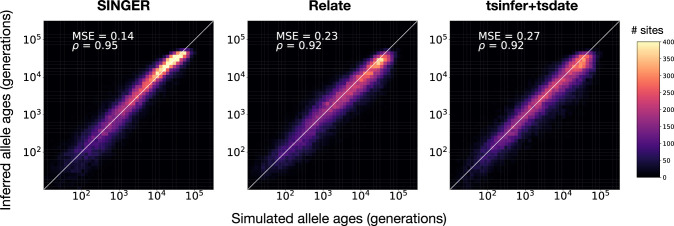
Inference of allele ages on 300 sequences. Performance of inferring allele ages with SINGER, Relate and tsinfer+tsdate, with 300 sequences. SINGER performs the best, followed by Relate, and tsinfer+tsdate performs the worst. ARG-Needle is excluded because it does not map mutations to branches of the inferred ARG.

We also benchmarked allele age estimation using inferred ARGs, excluding ARG-Needle and ARGweaver because ARG-Needle does not map mutations to branches, and ARGweaver’s output is difficult to parse for this task. On simulated data with 50 sequences, SINGER noticeably outperformed Relate and tsinfer + tsdate (Fig. 3a). For 300 sequences, SINGER remained more accurate than Relate and tsinfer + tsdate (Extended Data Fig. 2).

We also compared the number of recombination breakpoints in 5 kb windows. Only ARGweaver and SINGER produced accurate estimates (Fig. 3b). Both Relate and tsinfer missed many recombination events, consistent with earlier studies^37^.

Finally, we assessed the accuracy of recombination inference by the distribution of pairwise identity-by-descent (IBD) lengths, which are shaped by recombination. ARGweaver and Relate were excluded from this analysis; the former because of difficulties extracting IBD information from its output, and the latter owing to a lack of node persistence across marginal trees. As illustrated in Fig. 3c, SINGER accurately captured the distribution of pairwise IBD lengths, while tsinfer substantially overestimated IBD lengths, consistent with previous findings^37^.

#### Comparison of MCMC convergence

In humans and many other organisms, the genome-wide average rates of recombination and mutation are similar, which leads to substantial uncertainty in ARG inference. Therefore, it is important to obtain samples from the posterior distribution and characterize uncertainties, rather than relying on point estimates. To assess MCMC convergence, we obtained 100 posterior MCMC samples from ARGweaver, Relate and SINGER, using the same burn-in and thinning intervals.

**Fig. 4:**
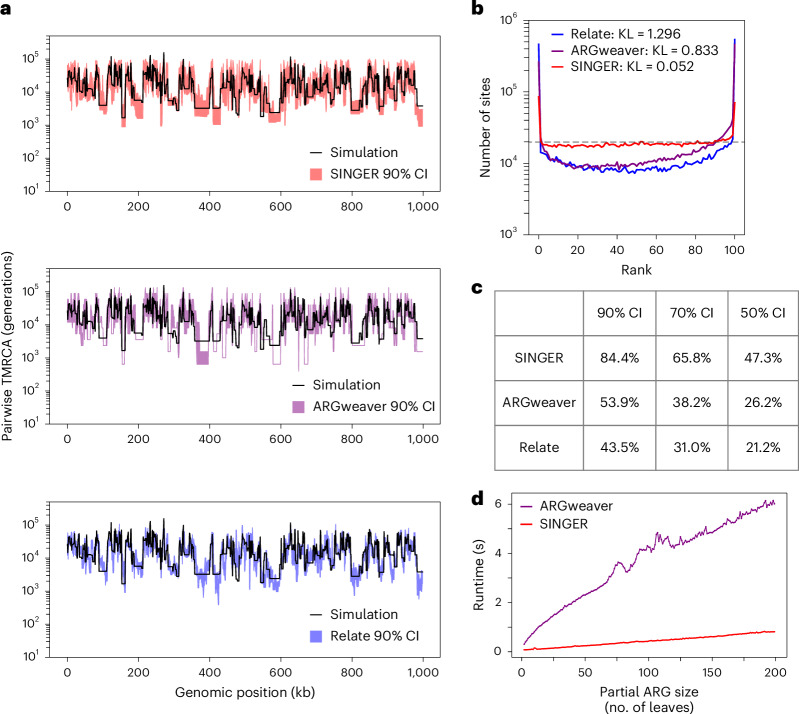
Properties of ARG samples and runtimes. **a**, Empirical 90% CIs for pairwise coalescence times as inferred by SINGER, ARGweaver and Relate. **b**, Rank plots of pairwise coalescence times in MCMC samples. A perfect sampler from the posterior distribution would achieve the flat dashed line, corresponding to the uniform distribution. The Kullback–Leibler (KL) divergence is used to quantify deviation from a uniform distribution. **c**, The empirical coverage of the ground truth pairwise coalescence time by the CI for different nominal levels. **d**, The runtime of the threading algorithm as a function of the partial ARG size (measured by the number of leaves), for ARGweaver and SINGER.

To assess the posterior sampling effectiveness, we used the same benchmark as in previous work^36^. This involved analyzing rank plots of pairwise coalescence times; a uniform distribution would be achieved by a perfect sampler from the posterior distribution^51,52^. A rank plot is a histogram of the rank of a parameter sampled from the prior relative to the posterior sample. Ideally, a converged and well-mixed MCMC should yield uniformly distributed ranks. By contrast, a U-shaped rank plot suggests sampling from an under-dispersed distribution^51,52^. Compared to ARGweaver and Relate, SINGER’s rank plots are much closer to the uniform distribution (Fig. 4b).

The rank plot is closely related to the coverage property of empirical credible intervals (CIs). For each genomic position and pair of haplotypes, the empirical 90% CI is defined by the 5^th^ to the 95^th^ percentile of the sampled coalescence times (Fig. 4a). The same approach was applied to the 70% and 50% CIs. The 90% CI covered the ground truth in only 44% of instances for Relate and 54% for ARGweaver. By contrast, the coverage was substantially better for SINGER, at 85% (Fig. 4c). SINGER also compared favorably at other CI levels (Fig. 4c).

**Extended Data Fig. 3:**
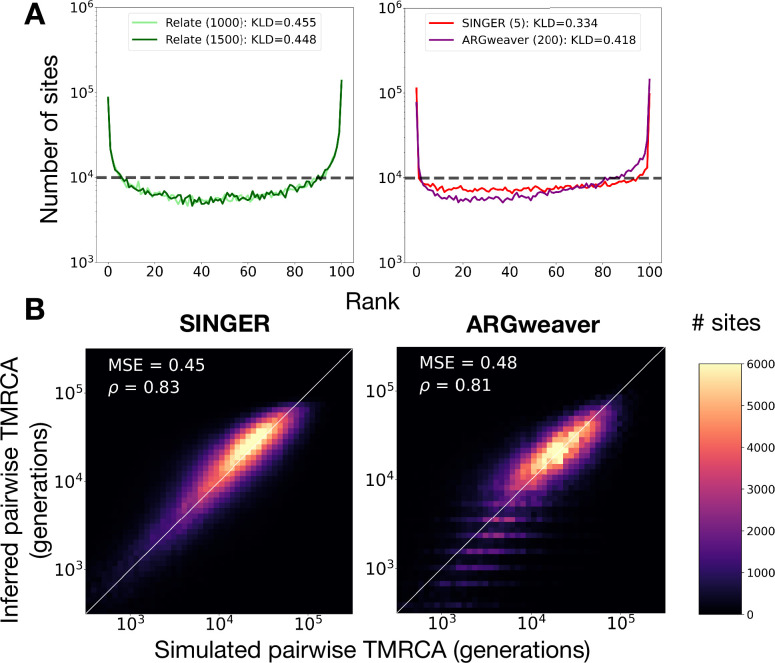
The influence of thinning on MCMC sampling. (**a**) Rank plots for different methods. In these results, considerably longer thinning intervals, indicated in parentheses, were used for ARGweaver and Relate. Relate is close to saturated thinning because changing the thinning interval from 1,000 to 1,500 results in little change in the rank plot. (**b**) Pairwise TMRCA inference from ARGweaver with thinning interval length 200 compared to that from SINGER with thinning interval length 5. ARGweaver still underperforms compared to SINGER in both rank plot and pairwise TMRCA, even with 40 times longer thinning than SINGER.

Furthermore, even with thinning intervals 40 times longer than SINGER, ARGweaver still underperforms in pairwise coalescence time inference and CI coverage (Extended Data Fig. 3). Combined with our faster threading algorithm, this suggests that ARGweaver would require hundreds to thousands of times longer to match SINGER’s performance. For Relate, even with long thinning intervals, CI coverage remains substantially below nominal levels (Extended Data Fig. 3 and Supplementary Section C.4), probably because Relate samples only coalescence times under a fixed topology, whereas SINGER samples both topologies and coalescence times.

#### Runtime comparison

Given that both SINGER and ARGweaver use threading algorithms, we compared their threading runtimes as a function of the number of leaves in the partial ARG. SINGER’s threading is approximately 10× faster than ARGweaver’s (Fig. 4d).

#### Other benchmarks

We performed additional benchmarks^53^ (Supplementary Sections C.2 and C.5 and Supplementary Fig. 7) and observed that SINGER outperforms other methods. We also note that a recent independent benchmarking study found that SINGER outperforms other ARG inference methods in reconstructing allele frequency trajectories and polygenic score histories^54^.

SINGER supports using an input recombination map to account for the recombination rate variation along the genome^55,56^, which improves inference accuracy (Supplementary Section C.6 and Supplementary Figs. 8 and 9).

### Applications to WGS data from the 1000 Genomes Project

We applied SINGER to 200 whole-genome sequences from five African indigenous populations (GWD, YRI, ESN, LWK and MSL) in the 1000 Genomes Project^57^, with 40 genomes randomly sampled per population (Supplementary Section D.1). To demonstrate the utility of SINGER, we analyzed population differentiation in coalescence times, trans-species polymorphism and archaic introgression. We also ran SINGER and Relate on the British (GBR) population data from the 1000 Genomes Project (Supplementary Section D.1) and used tsinfer + tsdate ARG from a previous publication^3^ for comparison.

#### Diagnostics of the ARGs sampled by SINGER

**Extended Data Fig. 4:**
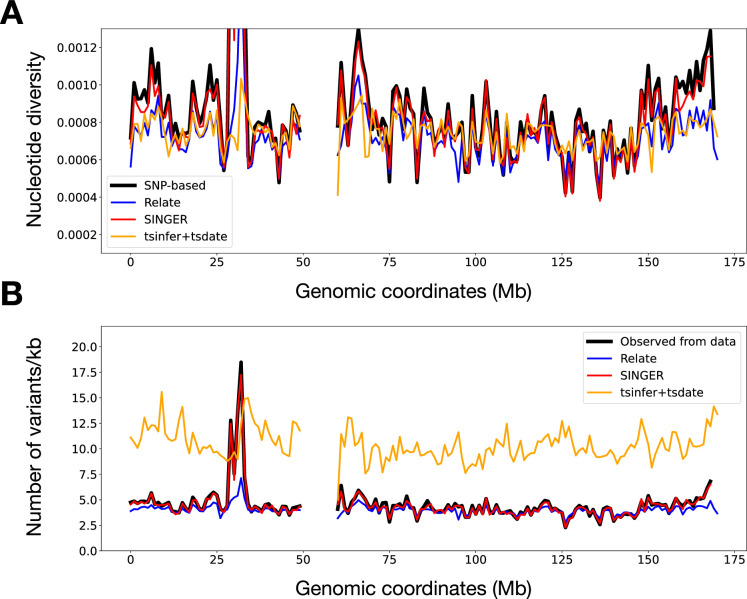
Consistency with Mb scale statistics. The 1~Mb nucleotide diversity (**a**) and variant density (**b**) landscape for chromosome 6 in GBR, computed from real data (black) versus inferred from Relate (blue), SINGER (red), and tsinfer+tsdate (orange).

We examined the sampled ARGs to check MCMC convergence and to ensure that sampling for inference occurred past proper burn-in (Supplementary Section D.4). The chains generally converged well (Supplementary Fig. 10). Additionally, we validated the accuracy of the sampled ARGs by comparing the inferred average pairwise coalescence times (scaled by $4{N}_{e}\mu$) with empirical single-nucleotide polymorphism (SNP)-based nucleotide diversities in 1 Mb windows, which showed high concordance. By contrast, Relate and tsinfer + tsdate^3^ underestimated the genome-wide variation of diversity (Extended Data Fig. 4a). This is possibly because of the ${N}_{e}$ variation as a result of background selection^58,59^. As shown earlier, SINGER is more robust to ${N}_{e}$ misspecification. Additionally, tsinfer + tsdate has a very biased variant density prediction from inferred ARG compared to observed data (Extended Data Fig. 4b). This is probably caused by polytomies, which distort the total branch length. In addition, Relate and tsinfer + tsdate require allele polarization (that is, distinguishing ancestral vs derived alleles), but it is difficult for the HLA locus with high levels of trans-species polymorphisms.

#### Population differentiation in coalescence times

**Extended Data Fig. 5:**
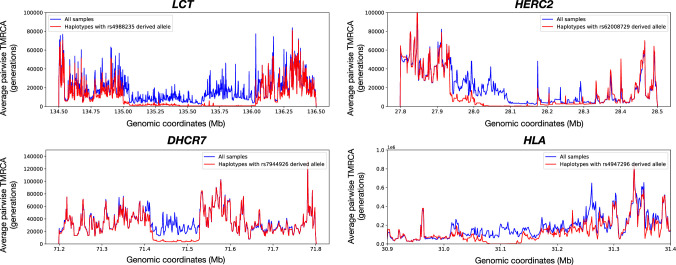
Examples of previously identified selection targets. The comparison of the average pairwise TMRCA among carriers of potential target SNP and all samples, for the candidate genes in the selection scan.

Population-level differentiation in coalescence times at the same genomic locus is often used to identify sites that warrant further evolutionary analysis^60–62^. Such differentiation could be a result of evolutionary forces such as local adaptations, which reduce diversity for the population experiencing selective sweeps. However, SNP-based diversity can be noisy at fine scales (Supplementary Fig. 11). On the other hand, with accurately inferred ARGs, fine-scale diversity can be estimated more accurately. We observed that SINGER produces more accurate estimates of fine-scale diversity than Relate and tsinfer + tsdate (Supplementary Section D.5 and Supplementary Fig. 11). This improvement facilitates studying population-specific fine-scale differentiation in coalescence times. Many previously reported loci under positive selection in Europeans^63^ appear as outliers when comparing the 1 kb-scale average pairwise time to the most recent common ancestor (TMRCA) between GWD and GBR (Supplementary Fig. 12), probably reflecting European-specific selection. They also exhibit long segments of reduced pairwise TMRCA among target allele carriers compared to the overall sample (Supplementary Section D.6 and Extended Data Fig. 5). Here, we focus on population-specific reduction in local diversity in African populations.

**Fig. 5:**
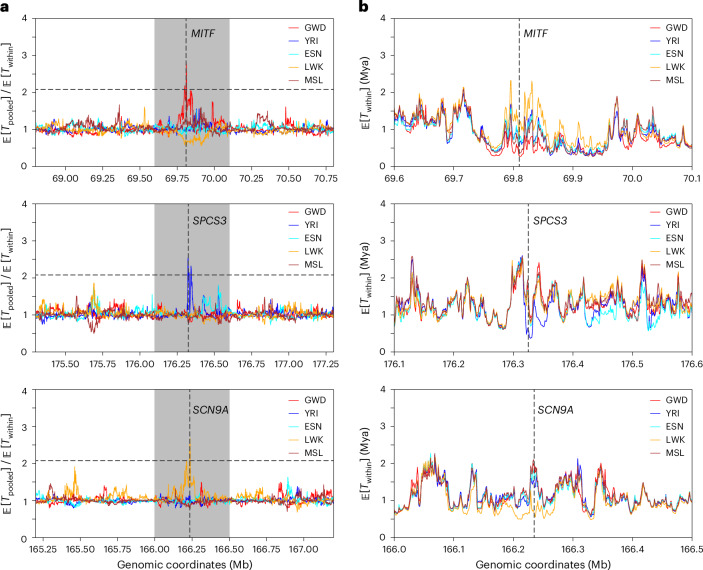
ARG-based detection of differentiated coalescence times in African populations. **a**, The ratio of the average pairwise coalescence time in the pooled sample, *T*_pooled_ (combining all five populations), to the average population-specific pairwise coalescence time, *T*_within_, for every 1 kb window. In each plot, the horizontal black dashed line denotes the genome-wide 99.99% quantile, and the gray shaded area corresponds to a 50 kb window surrounding the peak. The positions of these peaks are marked by vertical dashed lines, and the genes overlapping with these signals are indicated. **b**, The average *T*_within_ for each population, zoomed into the gray regions highlighted in **a**.

To find population-specific reduction in local diversity, we partitioned the genome into 1 kb windows and computed the ratio of the ARG-based diversity estimate for the combined sample to that for each of the five populations; reductions in local diversity would show up as peaks when these ratios are plotted along the genome. The full list of regions with elevated ratios for each population is available in the Data Availability section. We highlight a few interesting findings in Fig. 5 and Supplementary Fig. 13. For example, we found that the gene *MITF* has experienced a reduction in diversity in GWD relative to other populations (see also Supplementary Fig. 14); this gene has been reported to be related to skin^64^. Around *MITF*, we observed substantial differences in local diversity across the five populations, consistent with pigmentation variation within Africa^61^. In YRI, we found that *SPCS3* has reduced diversity in YRI compared to other populations; this gene encodes an immune-related protein believed to impact virion production of flaviviruses such as yellow fever virus^65^. This is concordant with the report of the spread of these diseases in Nigeria^66^. Lastly, we found that *SCN9A*, which encodes a voltage-gated sodium channel involved in the perception of pain^67^, has substantially reduced diversity. These examples illustrate the utility of SINGER for exploratory evolutionary analysis, but additional studies are needed to investigate if, and how, selection is acting in these loci.

#### Archaic introgression

**Fig. 6:**
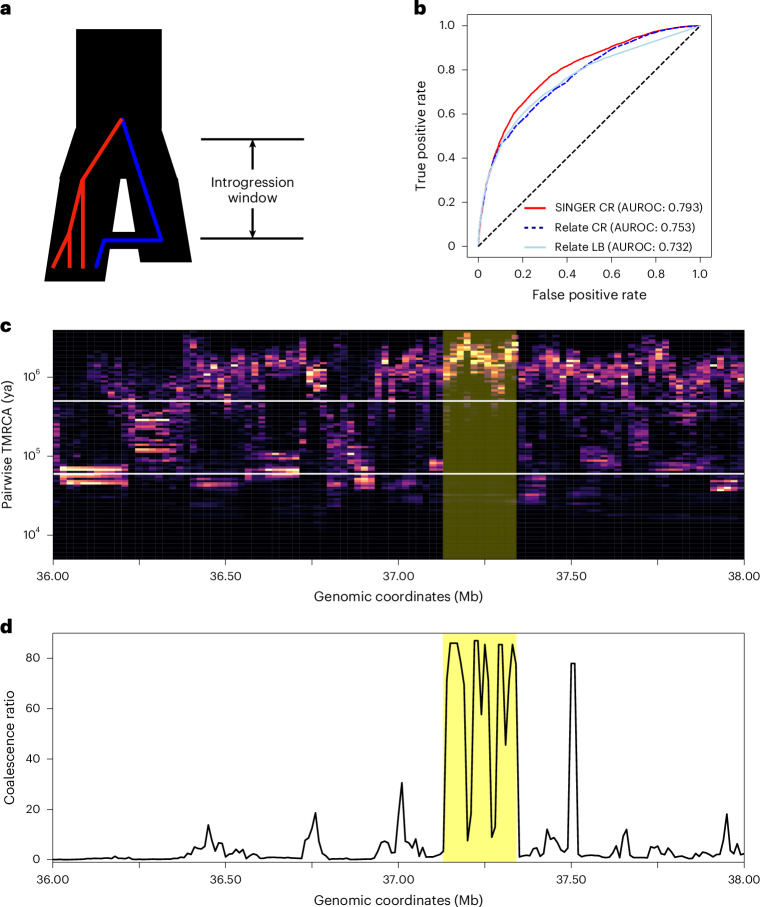
ARG-based detection of archaic introgression tracts. **a**, The demography model involving introgression and the un-introgressed (red) and introgressed (blue) lineages under the model. The time interval from the introgression time to the divergence time of the two populations is called the ‘introgression window’. **b**, The receiver operating characteristic (AUROC) plot of using coalescence ratio (CR) and long branch proportion (LB) from Relate and SINGER to differentiate the inferred introgressed and un-introgressed tracts from IBDmix. **c**, Identification of potential archaic introgression tracts. For a given leaf node, its pairwise coalescence times with every other leaf node in the marginal tree are summarized as a distribution. In the plot, each column represents such a distribution from marginal trees within a 10 kb window. The two white horizontal lines delineate the interval between the introgression time and the split time. A tract indicative of introgression should exhibit a depletion of coalescence events within this interval and an enrichment of coalescence events above the split time. Regions shaded in red denote putative introgression tracts. **d**, The ratio of pairwise coalescence density above the split time to that within the interval between the introgression time and the split time.

Evidence shows that modern humans carry DNA segments from Neanderthals and Denisovans^68–70^ as well as unidentified hominid groups^71^. Identification of these introgressed genomic tracts is a challenging task, especially when there is little or no known genome of the source hominids. However, ARGs can facilitate this task by the following observation: for an introgressed tract in a given haplotype, its coalescence with other haplotypes will be depleted in the interval between the introgression time and the split time of modern humans from the ‘ghost’ population (Fig. 6a). This is similar to the ‘long branch’ signals described in previous work^16^, but expressed in the pairwise coalescence space.

However, the ‘long branch’ signals can be sensitive to topology inference errors (Supplementary Fig. 15); specifically, the introgressed lineage can group incorrectly with the ancestral lineages of non-introgressed sequences, thereby destroying the long branch (Supplementary Section D.7). To mitigate this issue, we provide a technique based on the coalescence distribution heatmap. For each sequence, we plot the distribution of its pairwise coalescence time with the remaining sequences in 10 kb windows (Fig. 6c), in which each column corresponds to a 10 kb window. We found that using posterior samples of ARGs is helpful, as the coalescence distribution from a single ARG can be noisy (Supplementary Fig. 16). ARG samples with different topologies help smooth the heatmap (Supplementary Fig. 16). This is related to the visualization shown in previous work^48^.

To detect introgression tracts, we look for a depletion of probability mass in the aforementioned interval and an enrichment of mass above the interval. This is more robust than long branches because slight mis-grouping would still lead to probabilistic depletion in the interval, whereas the long branch would be disrupted completely. We demonstrated the feasibility of this approach by showing that the ARGs inferred by SINGER can recover the Neanderthal introgression tracts inferred by IBDmix^72^, a referenced-based method directly comparing Neanderthal and modern genomes (Supplementary Section D.7). The coalescence ratio slightly outperforms long branch signals for detecting archaic introgression in Relate, and SINGER compares favorably with Relate on this task (Fig. 6b).

Here, we highlight a potential 200 kb Neanderthal introgression tract in GBR (Fig. 6c), supported by IBDmix and the coalescence depletion signal from SINGER-inferred ARGs. We use 60 kya and 500 kya for introgression and split times, respectively, following previous work^73^, and plot the ratio of coalescence probability above the split time to that in the interval between introgression and split times. This tract appears as distinctive peaks in the ARG-based analysis (Fig. 6d).

#### Trans-species polymorphism in the HLA locus

The HLA locus comprises a cluster of genes that encode transmembrane proteins that present antigen peptides to T cells. This region is known to be the most diverse region in the human genome, and it has been hypothesized to be under extreme balancing selection to maintain high diversity to handle various immune challenges^74,75^. There has been evidence of trans-species polymorphism for some alleles across primates, which otherwise is very rare^74^.

**Fig. 7:**
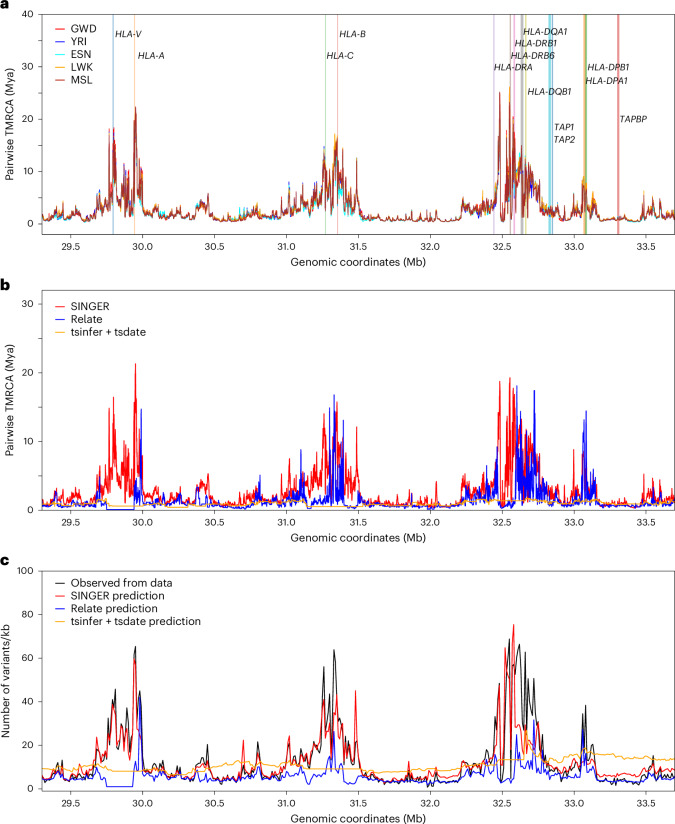
The fine-scale diversity landscape in the HLA region in Africans and GBR, and comparison between observed and inferred mutation density in GBR. **a**, The average pairwise coalescence time in the HLA in the African sample, with a few genes highlighted (vertical bar colors have no meaning aside from denoting different genes). **b**, The average pairwise coalescence time in the HLA in GBR, inferred from SINGER, Relate and tsinfer + tsdate. **c**, The observed mutation density from data compared with that predicted from ARGs, inferred from SINGER, Relate and tsinfer + tsdate.

The ARGs inferred by SINGER show extremely ancient pairwise coalescence times in the HLA locus, with many regions harboring coalescence times older than the human–chimpanzee divergence time (Fig. 7a,b). In African individuals, we computed the average TMRCA in 1 kb windows on chromosome 6 and found that HLA is the only region with the average TMRCA above 10 million years (Supplementary Fig. 17), making erroneous ARG inference an unlikely explanation for these ancient coalescence times. This is consistent with the hypothesis of strong balancing selection in this locus and the known trans-species polymorphisms. The human–chimpanzee divergence time is estimated to be 5–12 Mya^76^. Although many genes in the HLA region do not show strong evidence of coalescence times older than the human–chimpanzee split (for example, *TAP1, TAP2* and *TAPBP*), many do, including *HLA-A*, *HLA-DRB1* and *HLA-DRB6*. Unsurprisingly, there are no noticeable differences across the five populations, as the polymorphism has been maintained since ancient times. By contrast, in GBR, Relate and tsinfer + tsdate^3^ do not recover such extreme coalescence times (Fig. 7b), probably owing to poor allele polarization in the HLA locus and model misspecification arising from deviations from selective neutrality. To validate our results, we compared the mutation densities in 10 kb windows from real data with predictions from the inferred ARG; SINGER provides a good fit, while Relate and tsinfer + tsdate underestimate substantially (Fig. 7c).

In addition to the HLA locus, we extended the analysis genome-wide to find other loci with exceptionally ancient coalescence times (Supplementary Fig. 18), some of which coincide with previous findings of long-term balancing selection, including *TRIM5* (ref. ^77^), *ABO*^78^, *IGFBP7* (ref. ^79^), *PKD1L1* and *DMBT1* (ref. ^80^).

## Discussion

In this article, we introduced SINGER, a Bayesian method for efficiently sampling ARGs from the posterior distribution. SINGER implements an improved MCMC algorithm to explore the ARG space, thereby enabling accurate uncertainty characterization in both coalescence times and ARG topologies. Our approach scales to at least hundreds of whole-genome sequences while performing full posterior sampling of both ARG branch lengths and topologies. Compared to ARGweaver, SINGER uses faster threading and more efficient MCMC algorithms. In estimating key population genetic quantities—such as coalescence times, topologies, recombination densities and allele ages—SINGER compares favorably with existing methods, including ARGweaver, Relate, tsinfer + tsdate and ARG-Needle. As demonstrated in our benchmarks, using posterior samples can noticeably enhance inference accuracy and effectively quantify estimation uncertainty. Last but not least, SINGER exhibits greater robustness to model misspecification, such as population size changes and background selection.

We applied SINGER to data from individuals of British descent and individuals of African descent from the 1000 Genomes Project. Using ARG-based fine-scale diversity estimates, we identified genomic regions with exceptional population differentiation in coalescence times across African populations. In addition, we used a visualization technique, the coalescence distribution heatmap, to identify genomic regions consistent with a specific model of archaic introgression. Lastly, we found strong evidence of trans-species polymorphisms in the HLA region and mapped genes associated with the peaks in these signals.

We note that our proposed approach for detecting archaic introgressed tracts requires a demographic model. However, there is ongoing debate regarding the timing, strength and even the existence of certain archaic introgression^81^. In this regard, the tract identification requires reasonably accurate introgression and split times. Moreover, the detection of introgressed tracts using sampled ARGs warrants further methodological development. Our proposed heatmap of coalescence distribution provides a basis for future methods.

Despite its strength, SINGER has some limitations and room for improvement. First, although it is substantially more scalable than alternative methods for posterior sampling of ARGs, real data applications often require a large number of MCMC iterations. More efficient ARG exploration strategies are therefore needed.

Second, although SINGER shows improved robustness to model misspecification compared to other methods, its inference accuracy might be further enhanced by jointly inferring population size history and branch lengths, similar to the approach used by Relate^16^. However, Relate’s algorithm for inferring population size history and branch lengths cannot be readily applied to other ARG inference methods. On the other hand, tsdate is compatible with SINGER’s data structure but assumes a constant population size. More generally, SINGER could be extended to incorporate complex demographic models, as in ARGweaver-D^82^.

Third, SINGER may not be suitable for certain data. For example, it assumes an infinite-sites model, which may not be applicable to cross-species data. Incorporating a finite-sites model, as in ARGweaver^15^, could help. SINGER also requires WGS data and cannot analyze genotyping arrays directly. The locus-skipping algorithm^49,83^ can facilitate the analysis of SNP genotyping array data, but applying ARG-Needle directly to genotype array data substantially reduces accuracy (Supplementary Section E.1 and Supplementary Fig. 19). By contrast, imputing genotypes using a reference panel before applying SINGER yields higher accuracy (Supplementary Fig. 19). Therefore, when genome imputation is feasible, it may be preferable to the use of the locus-skipping algorithm.

Lastly, SINGER requires phased, contemporary genomes. Unfortunately, high-quality phasing is often challenging, especially for ancient DNA and non-model organisms. Therefore, supporting unphased data would increase SINGER’s utility, especially for joint analysis of ancient and modern genomes, which are often poorly phased or completely unphased.

## Methods

### Branch sampling

To speed up the computation, we first partition the genome into equal-sized bins, in which the bin size is chosen to be about $4\times {10}^{-3}/(4{N}_{e}r)$. We then construct an HMM indexed by these bins (loci) to sample the branches at which the lineage for the *n*^th^ haplotype joins the partial ARG for the first *n* − 1 haplotypes. The state space ${S}_{l}$ for bin $l$ comprises all the branches in the marginal tree for bin $l$ in the partial ARG and some branches from earlier bins. The precise definition of the state space can be found in Supplementary Section B.1. For bin $l$, we use ${B}_{l}$ to denote the branch onto which the lineage for the *n*^th^ haplotype joins. If the partial ARG does not already contain a recombination event between bins *l* − 1 and *l*, then the transition probability of the HMM is defined as:

$$P\left({B}_{l}={b}_{j}|{B}_{l-1}={b}_{i}\right)=\left(1-{r}_{i}\right){{\rm{\delta }}}_{{ij}}+{r}_{i}{q}_{j}/\left(\sum _{k:{{\rm{b}}}_{k}\in {S}_{l}}{q}_{k}\right)$$

where ${b}_{i}\in {S}_{l-1}$, ${b}_{j}\in {S}_{l}$ and ${r}_{i}$ denotes the branch-specific recombination probability for branch ${b}_{i}$. The definition of ${r}_{i}$ and ${q}_{j}$, which are computed under the assumption of a constant-sized panmictic population, can be found in Supplementary Section B.1.5. The structure of this transition probability is similar to that of the Li–Stephens model^17^, but with branch-specific recombination and re-joining probabilities. This allows us to reduce the HMM computational complexity to be linear with respect to the number of hidden states, as in the Li–Stephens model.

We restrict the final ARG to have at most one recombination event between adjacent bins. Therefore, if the partial ARG already contains a recombination event between bins $l-1$ and $l$, the threading operation is not allowed to introduce an additional recombination between these two bins, the transition probability in this case is defined similarly to that in previous work^15^; the details are provided in Supplementary Section B.1.4 and Supplementary Fig. 30.

### Time sampling

Conditioned on a sequence of joining branches along the genome resulting from the branch sampling algorithm, the time sampling step proceeds in a similar fashion as in (pairwise) PSMC^12^ for a constant-sized panmictic population, but with the restriction that for each bin $l$, the coalescence time should reside between the two endpoints of the joining branch for bin $l$. To accelerate the computation, we implemented a previously published linearization technique^84^. The details are provided in Supplementary Section B.2.

### ARG re-scaling

**Extended Data Fig. 6:**
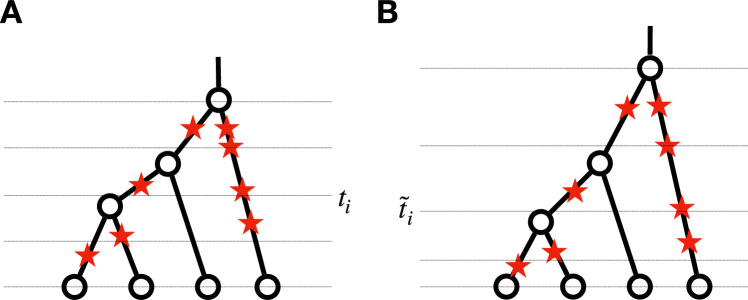
ARG rescaling from original ARG (a) to a new ARG (b). For simplicity we only show a single tree. We partition the ARG and count the number of mutations in each interval, and re-scale the interval length so that the expected number of mutations match the observed mutation counts. For example, the highest interval has more mutations mapped to it than the lowest interval, so it is widened and the latter is narrowed.

Given an inferred ARG, we partition the time axis into non-overlapping windows such that the total branch length across all marginal trees (weighted by the span of each tree) in each time window is the same; by default, 100 windows are chosen. We then count the number of mutations falling into each of these windows. If a mutation falls on a branch striding multiple windows, then its contribution to the mutation count for each window is given by the proportion of the branch that overlaps with the window. We re-scale each window size such that the expected number of mutations for the window matches the empirical count (Extended Data Fig. 6). This is essentially a window-specific re-scaling to better match the mutation clock. Further details can be found in Supplementary Section B.3. ARG re-scaling is performed after the initialization and every thinning step.

### SGPR

In the MCMC algorithm, we propose updates to the current ARG by first removing some branches following a cut and then re-grafting from the breakpoint.

**Extended Data Fig. 7:**
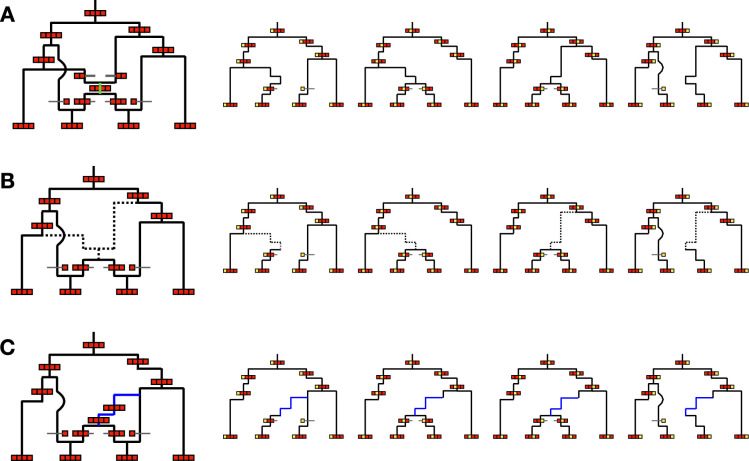
The connection between the Kuhner move and SGPR. ARG can be represented as the temporal network (left) or the spatial series of trees (right). (**a**) is the ARG before update in temporal network and spatial trees. In (**b**), the Kuhner move first picks a random branch (shown in green) in the network and removes any recombination or coalescence above from that branch, which is marked with dashed line (left). It is equivalent to choose a cut and trace it to the flanking region in SGPR (right). The partial branches connecting the cut to its upper node in every marginal tree is removed. In (**c**), the sub-graph has been re-grafted back with a new coalescent history, leading to potential changes in topology and branch length. For example, the recombination separating the second and third tree in the previous ARG (**a**) is gone.

To remove a branch from a given marginal tree, we make a random cut on the tree; the probability that a given branch will be cut is proportional to its length. We can extend the cut leftwards and rightwards along the genome, removing the partial branch from the cut to its upper endpoint of the branch. Typically, the cut will not extend over the entire chromosome; rather, the extension width will be the same as the span of the ancestral segment corresponding to the branch that was cut. The details can be found in Supplementary Section B.4 and Extended Data Fig. 7.

To re-graft the branch from the breakpoint, we use the same threading algorithm described in the Branch Sampling and Time Sampling sections above, with the only difference being that now we only consider the sub-ARG above the breakpoint. We show in Supplementary Section B.4.3 that, assuming the threading algorithm samples approximately from the posterior, the acceptance probability is typically much higher than that of previous proposals^15,41^, thereby improving convergence and mixing in MCMC.

### Simulation and benchmarking details

All coalescent simulations in this article were carried out using msprime^46^ with $r=\mu =2\times {10}^{-8}$ and ${N}_{e}=1\times {10}^{4}$. We simulated 50 datasets, each with 50 sequences over a 1 Mb region, and ten datasets, each with 300 sequences over 1 Mb. For simulations with 50 sequences, we also simulated with CEU population size history https://github.com/PalamaraLab/ASMC_data/tree/main/demographies estimated from SMC++ (ref. ^50^). We ran all inference methods with these true parameter values.

For Relate and tsinfer + tsdate, we use the posterior averages on their fixed estimated topology. ARG-Needle uses posterior averages of joining times when threading. SINGER and ARGweaver sample from the posterior over topologies, and we use 100 sampled ARGs for both.

As for MCMC sampling, we uniformized the number of iterations and the thinning scheme across all methods. We drew 100 samples with the thinning interval set to 20 for ARGweaver, Relate and SINGER, and used 1,000 iterations for burn-in.

For all simulation benchmarks involving ARGweaver and SINGER, posterior averages were taken for the statistics of interest, such as pairwise coalescence time, allele age and so on. Given that Relate outputs averages of MCMC iterations while tsdate results are averages from a probability table, we simply use their results from a single output, as they are effectively posterior averages.

### ARG inference methods benchmarked in this study

The version or the last-shown maintenance date of the ARG inference methods we considered in this study were as follows: SINGER (v.0.1.8), Relate (v.1.1.9), tsinfer (v.0.2.1), tsdate (v.0.1.4), ARG-Needle (March 2024) and ARGweaver (Jan 2017). We note that the code for a newer version (v.0.2.1) of tsdate was released while the manuscript was under review, but it is unpublished work and therefore we did not use it in our study.

### Reporting summary

Further information on research design is available in the Nature Portfolio Reporting Summary linked to this article.

## Online content

Any methods, additional references, Nature Portfolio reporting summaries, source data, extended data, supplementary information, acknowledgements, peer review information; details of author contributions and competing interests; and statements of data and code availability are available at 10.1038/s41588-025-02317-9.

## Supplementary information

Supplementary Information

Reporting Summary

## Data Availability

We have uploaded the inferred ARG samples (100 samples) and genes with exceptional population differentiation in coalescence times to Zenodo at 10.5281/zenodo.10437053 (ref. ^86^), 10.5281/zenodo.10467284 (ref. ^87^), 10.5281/zenodo.10467509 (ref. ^88^) and https://zenodo.org/records/10828414 (ref. ^89^).

## Code availability

SINGER is written in C++ with Python helper scripts. The source code can be downloaded from https://github.com/popgenmethods/SINGER (ref. ^85^).
