# Author Correction: Robust and accurate Bayesian inference of genome-wide genealogies for hundreds of genomes

**DOI:** 10.1038/s41588-025-02399-5

**Published:** 2025-10-10

**Authors:** Yun Deng, Rasmus Nielsen, Yun S. Song

**Affiliations:** 1 Center for Computational Biology, University of California, Berkeley, CA USA; 2 Department of Statistics, University of California, Berkeley, CA USA; 3 Department of Integrative Biology, University of California, Berkeley, CA USA; 4 Center for GeoGenetics, University of Copenhagen, Copenhagen, Denmark; 5 Computer Science Division, University of California, Berkeley, CA USA

**Keywords:** Population genetics, Software

Correction to: *Nature Genetics* 10.1038/s41588-025-02317-9, published online 8 September 2025.

In the version of this article originally published, there were labeling errors in several figures. In Fig. 2a, b, in the *y*-axis label now reading “Inferred pairwise TMRCA (generations),” the word “generations” was missing, while in the right-hand panel of Fig. 2a, the m.s.e. value now reading “0.78” appeared originally as “078.” In Fig. 3a, panel headers “SINGER; Relate; tsinfer+tsdate” were missing. The color keys in Fig. 4a were all mistakenly labeled “SINGER 90% CI” and now specify the ARGweaver and Relate methods. In Extended Data Fig. 3b, panel headers for the SINGER and ARGweaver plots were missing. The figures have been amended in the HMTL and PDF versions of the article.

