# Supplementary material for: Robust and accurate Bayesian inference of genome-wide genealogies for hundreds of genomes
Source: Nat Genet. 2025 Sep 8;57(9):2124–35. doi: 10.1038/s41588-025-02317-9 (PMC12425808; doi:10.1038/s41588-025-02317-9)
Supplement: Supplementary file 1 — Supplementary Information [file 41588_2025_2317_MOESM1_ESM.pdf]

nature genetics

Supplementary information https://doi.org/10.1038/s41588-025-02317-9

Robust and accurate Bayesian inference of genome-wide genealogies for hundreds of genomes

In the format provided by the authors and unedited

Contents

A Supplementary Figures **2**

B Method details **13**
- B.1 Branch sampling . . . 13
  - B.1.1 Representative joining time and joining probability for branches . . . 13
  - B.1.2 Deterministic approximation . . . 14
  - B.1.3 Emission probability . . . 14
  - B.1.4 Transitions induced by an existing recombination in the partial ARG . . . 15
  - B.1.5 Transitions with a new recombination . . . 16
- B.2 Time sampling . . . 18
  - B.2.1 Transition and emission probabilities . . . 18
  - B.2.2 Linearization of the forward algorithm for type A transitions . . . 19
  - B.2.3 Type B and type C transitions . . . 21
  - B.2.4 Inference of recombination times . . . 21
- B.3 ARG rescaling . . . 22
  - B.3.1 ARG rescaling with constant mutation rate . . . 22
  - B.3.2 ARG rescaling with mutation rate variation . . . 22
- B.4 Sub-Graph Pruning and Regrafting (SGPR) . . . 23
  - B.4.1 How to prune a sub-graph from an ARG . . . 23
  - B.4.2 Comparison of SGPR and the Kuhner move . . . 24
  - B.4.3 How to regraft the sub-graph to generate an updated ARG . . . 25
  - B.4.4 Metropolis-Hastings acceptance rate comparison between SINGER and ARG-weaver . . . 26
  - B.4.5 Runtime of SINGER . . . 27

C Simulation benchmarks **27**
- C.1 Comparison with PSMC in inferring pairwise TMRCA . . . 27
- C.2 Unscaled total variation distance . . . 27
- C.3 Robustness to model misspecification . . . 28
- C.4 The impact of thinning on rank plots . . . 28
- C.5 Expected site frequency spectrum . . . 28
- C.6 Recombination rate heterogeneity . . . 29

D Applications to the 1000 Genomes Project **29**
- D.1 Data and parameters for running ARG inference methods . . . 29
- D.2 Storage efficiency and the runtime of computing relevant statistics from inferred ARGs 29
- D.3 Large-scale diversity patterns . . . 30
- D.4 Convergence diagnostic of SINGER . . . 30
- D.5 Fine-scale diversity estimation . . . 31
- D.6 Previously reported selection targets in Britain . . . 31
- D.7 Introgression analysis . . . 32
- D.8 Other loci with ancient coalescence time . . . 33

E Additional Supplementary Information **34**
- E.1 Array data . . . 34

A Supplementary Figures

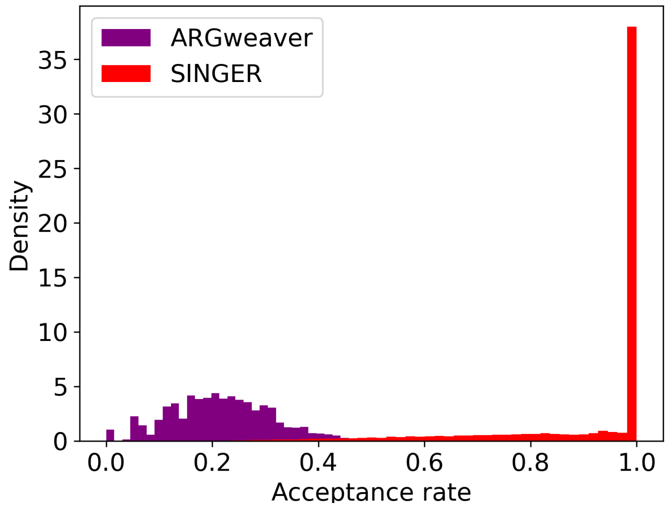

Supplementary Figure 1: The comparison of the acceptance rate distribution in MCMC in ARG-weaver (purple) and SINGER (red), with 50 sequences.

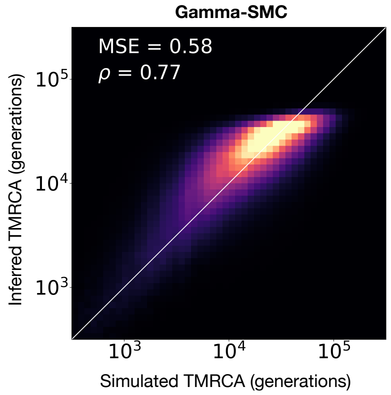

Supplementary Figure 2: Performance of Gamma-SMC, an ultra-fast pairwise coalescent method, on inferring pairwise TMRCA for 50 sequences. The performance is similar to that of Relate, but worse than SINGER.

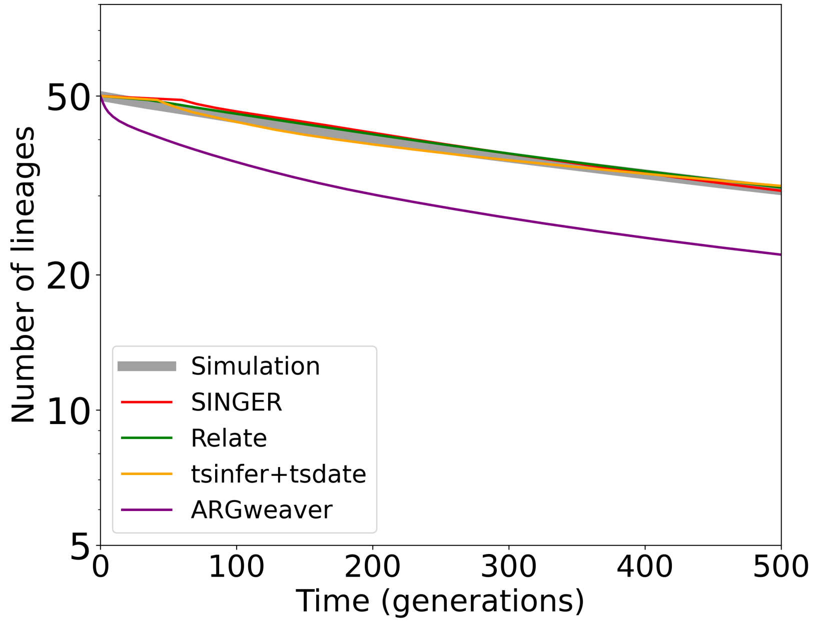

Supplementary Figure 3: A zoomed-in version of Figure 2D for $[0, 500]$ generations ago.

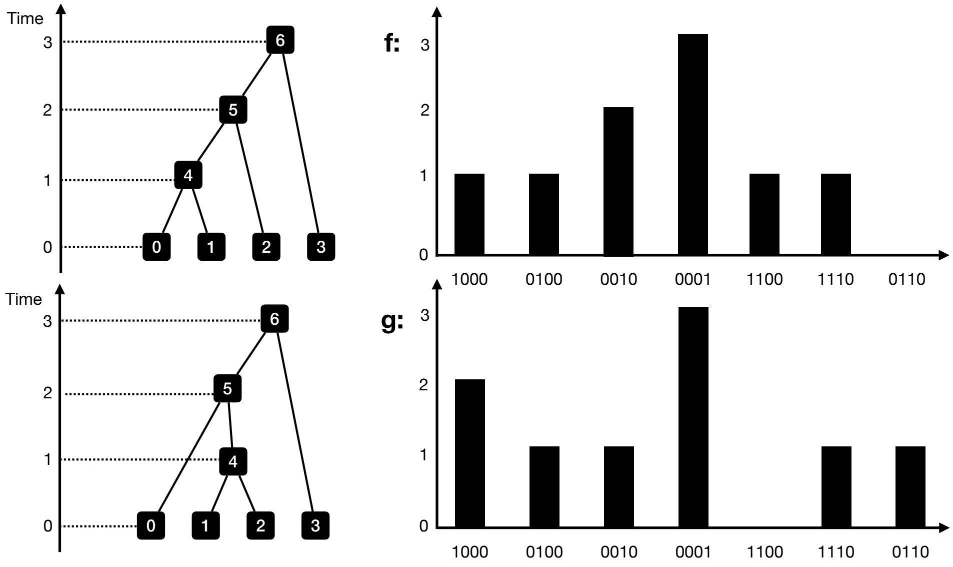

Supplementary Figure 4: An example of calculating the unscaled total variation distance for two coalescent trees. The unscaled total variation distance is calculated as $|f(1000)-g(1000)|+|f(0100)-g(0100)|+|f(0010)-g(0010)|+|f(0001)-g(0001)|+|f(1100)-g(1100)|+|f(1110)-g(1110)|+|f(0110)-g(0110)|=5$.

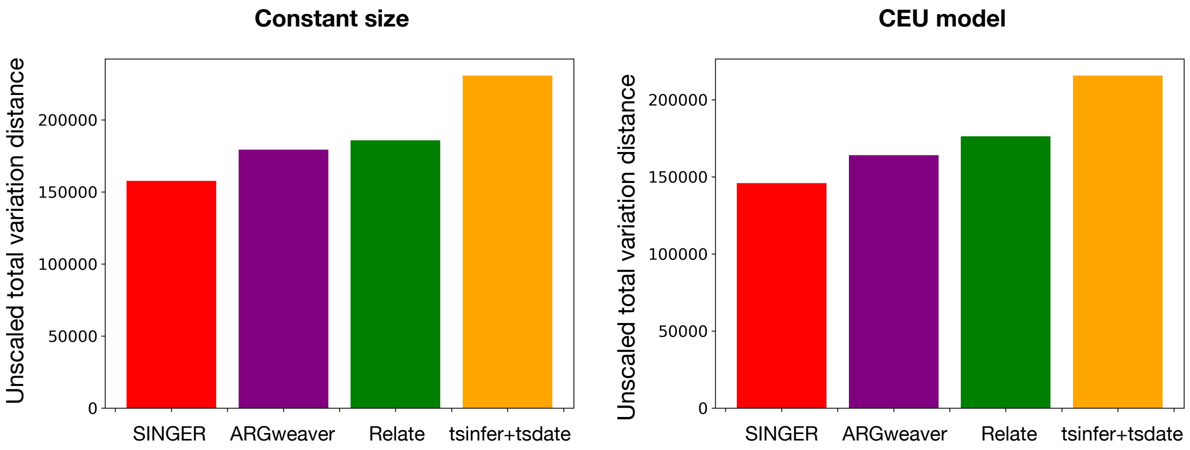

Supplementary Figure 5: The unscaled total variation distance between inferred ARGs and the ground truth ARG from simulation with 50 sequences, under a constant population size model (left) and the CEU model (right).

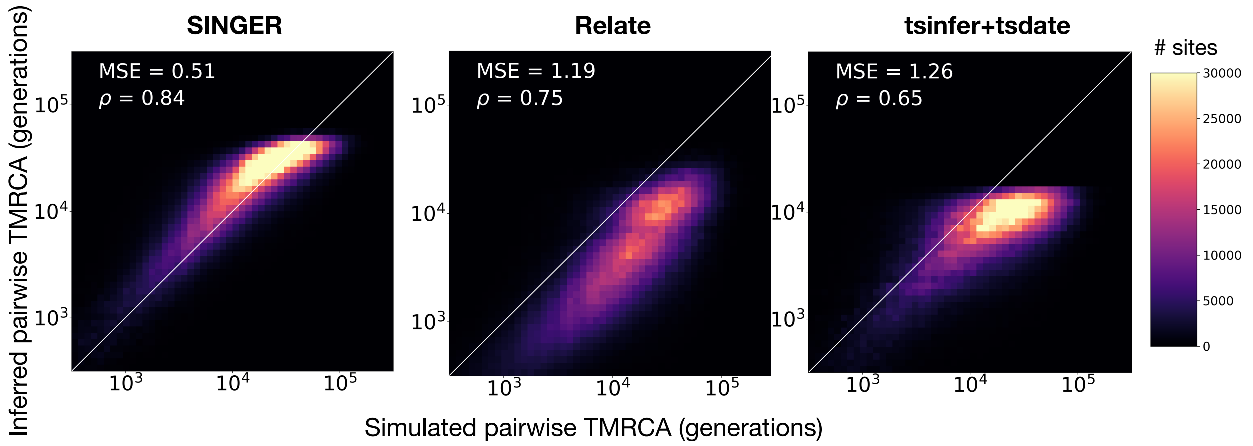

Supplementary Figure 6: Robustness of ARG inference methods, when data is simulated with $N_e = 10,000$ but ARG is inferred with $N_e = 2,000$.

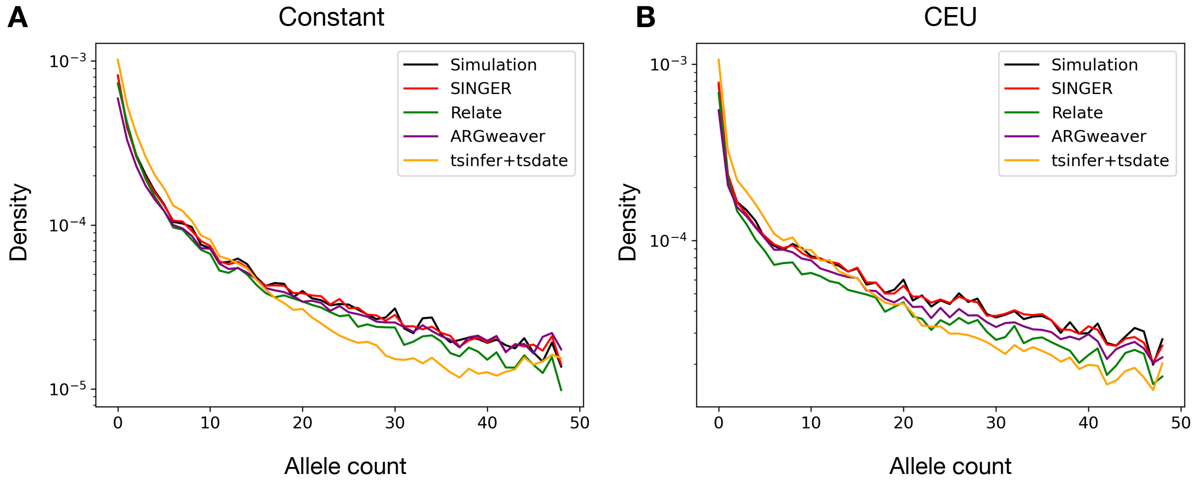

Supplementary Figure 7: Comparison of the expected SFS from the inferred ARG versus the observed SFS in the simulated data, for both a constant population size demographic model (A) and the CEU model (B).

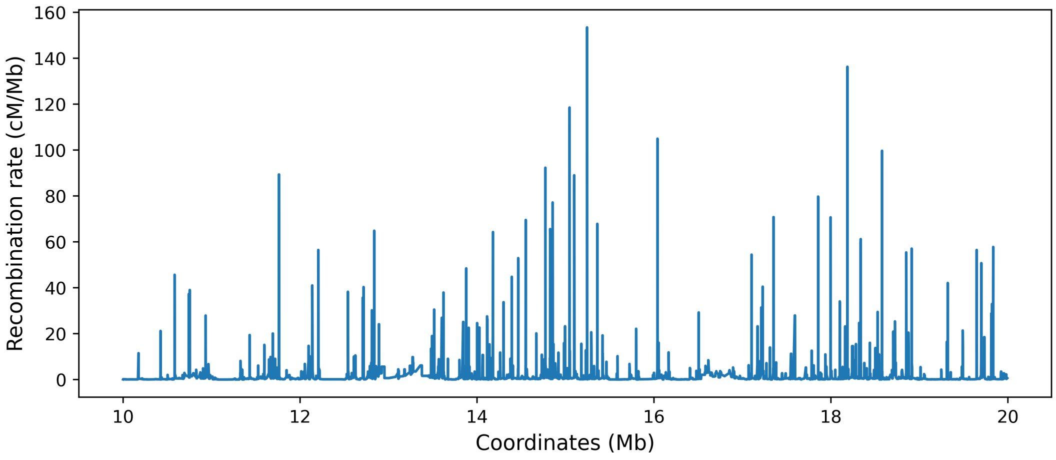

Supplementary Figure 8: The recombination map used in our coalescent simulations.

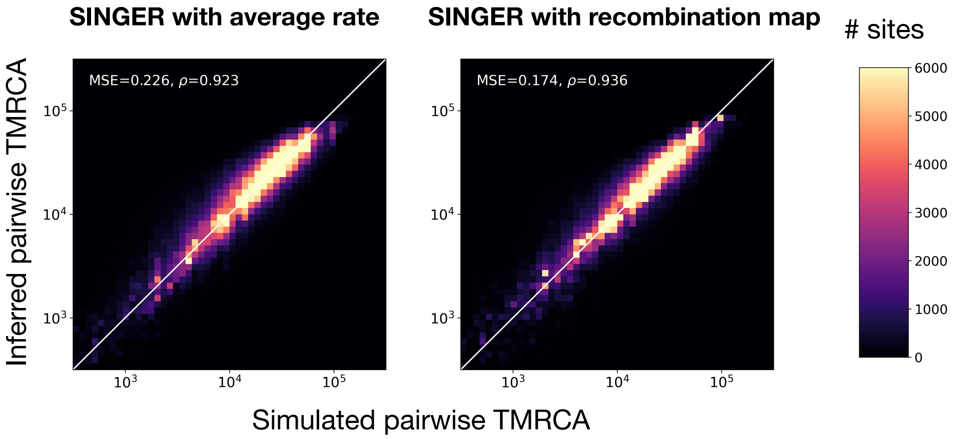

Supplementary Figure 9: The performance of pairwise TMRCA inference when running SINGER with the average recombination rate (left) or the fine-scale recombination map (right).

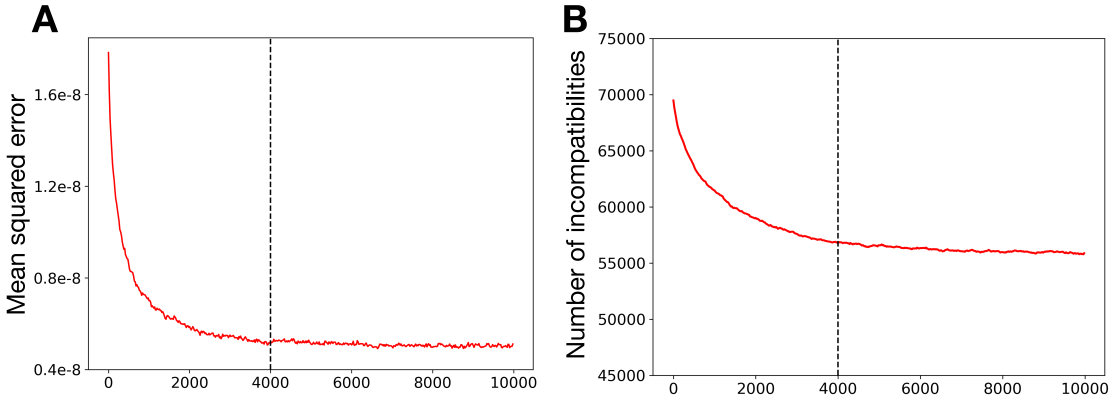

Supplementary Figure 10: (A) A trace plot of deviation (measured in MSE) in diversity variations. (B) The number of incompatibilities for SINGER samples of Chr2 for the African sample. We used a burn-in of 4,000 iterations, when the chain appears to have reached stationarity.

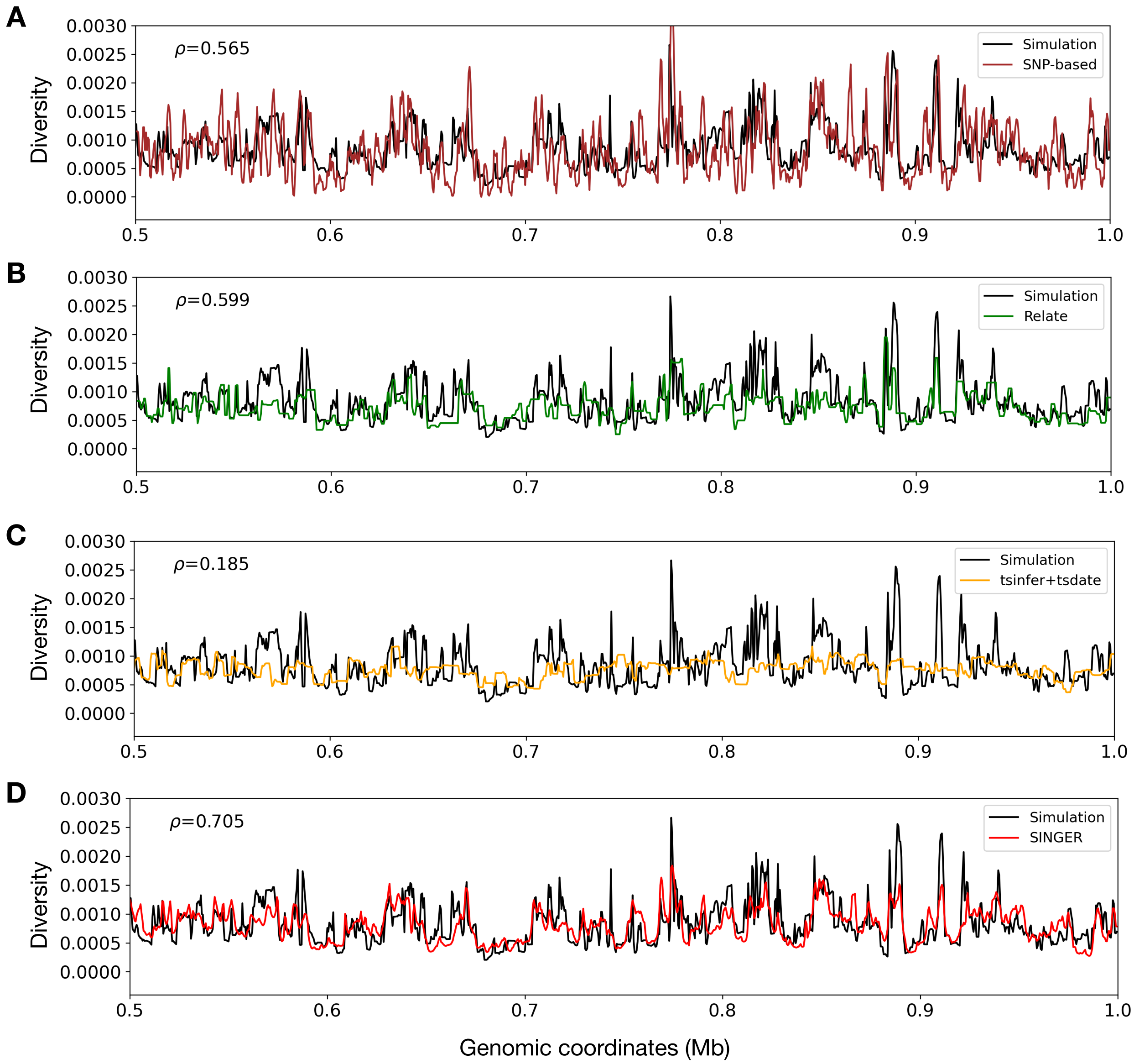

Supplementary Figure 11: Comparison of 500 bp fine-scale diversity estimates against the simulated ground truth. (A) SNP-based sliding windows. (B) Relate. (C) tsinfer+tsdate. (D) SINGER.

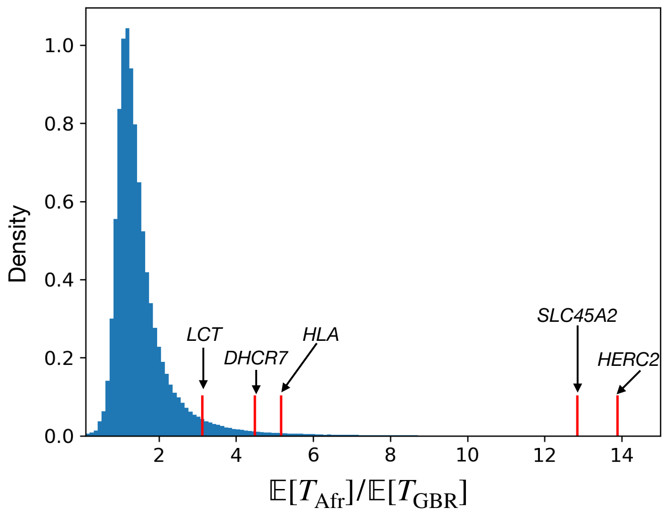

Supplementary Figure 12: The genome-wide distribution of the ratio of 1kb average pairwise TMRCA in Africans and GBR, and the positive selection target genes in Britain are marked with red lines.

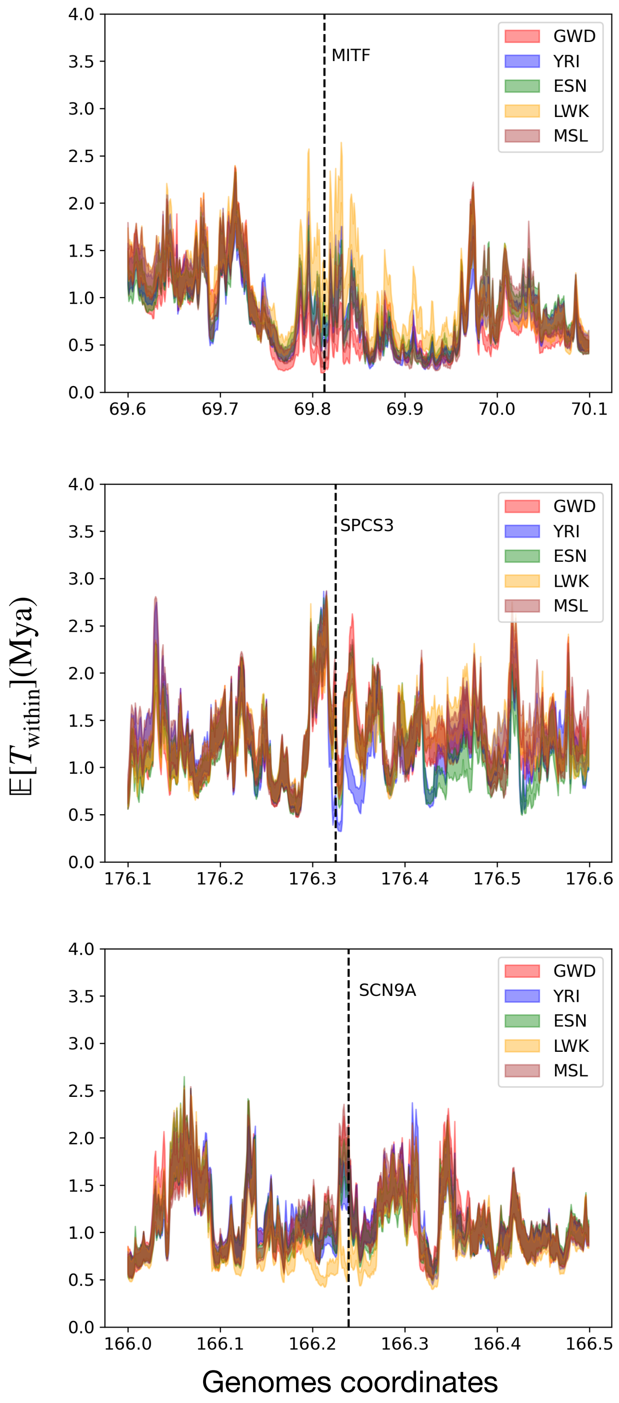

Supplementary Figure 13: The average T_{within} for each population, plotted with the 25% to 75% quantile credible interval from the posterior samples for the same regions in Figure 5.

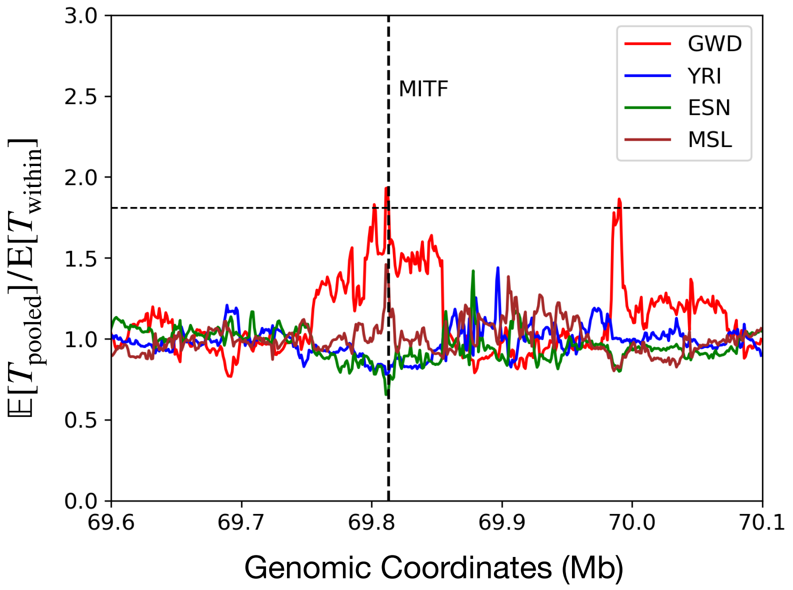

Supplementary Figure 14: The ratio of the average pairwise coalescence time T_{pooled} in the pooled sample (combining all five populations) to the average population-specific pairwise coalescence time T_{within}, when excluding LWK near the *MITF* region. The horizontal line is the new 99.99% quantile for chromosome 3.

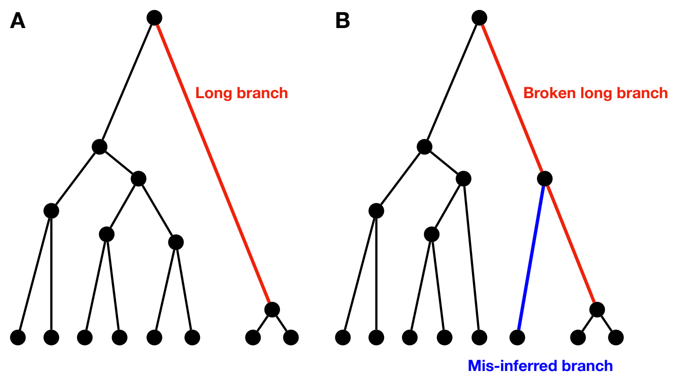

Supplementary Figure 15: The long branch resulting from introgression in a perfectly inferred local tree (A) and how it can be broken by incorrect topology inference (B).

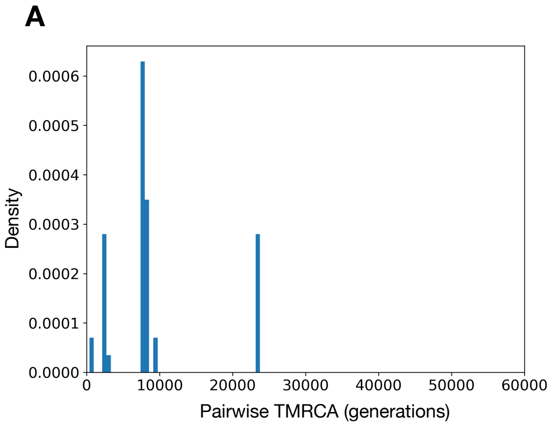

Supplementary Figure 16: The coalescence distribution in a single marginal tree, when using a singer tree estimate (A) versus a collection of posterior samples (B) with different branch lengths and topologies. By sampling the branch length and topology uncertainties, we are able to obtain a much more stable distribution.

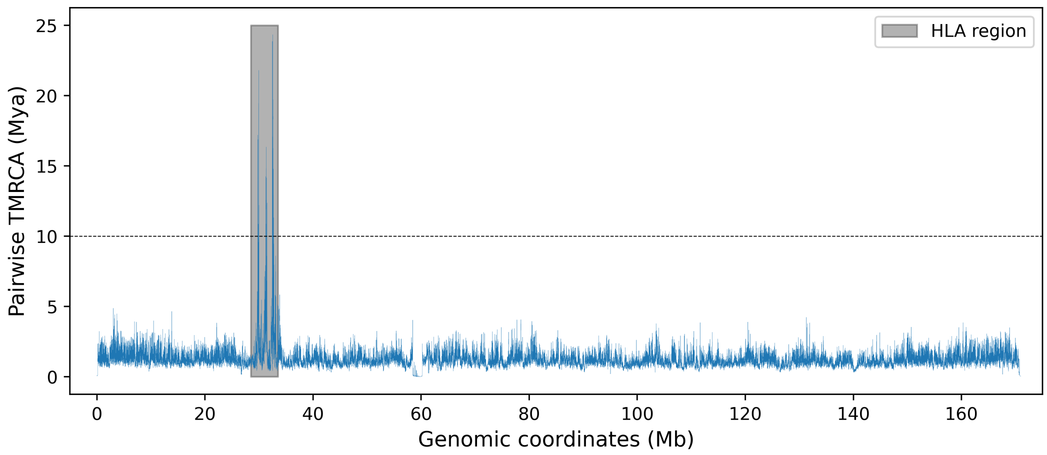

Supplementary Figure 17: Average pairwise TMRCA in Africans inferred by SINGER for chromosome 6. HLA is the only region (shaded in gray) that has pairwise TMRCAs substantially higher than 10 million years (black dashed line).

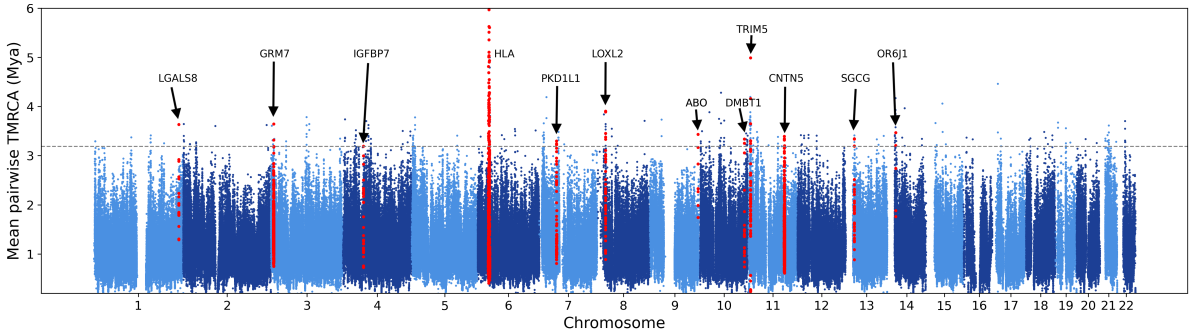

Supplementary Figure 18: A genome-wide scan for loci with exceptionally ancient coalescence times, using average pairwise TMRCA for 1kb windows. The dashed line is at around 3.2 Mya, the 99.99% quantile in non-HLA regions.

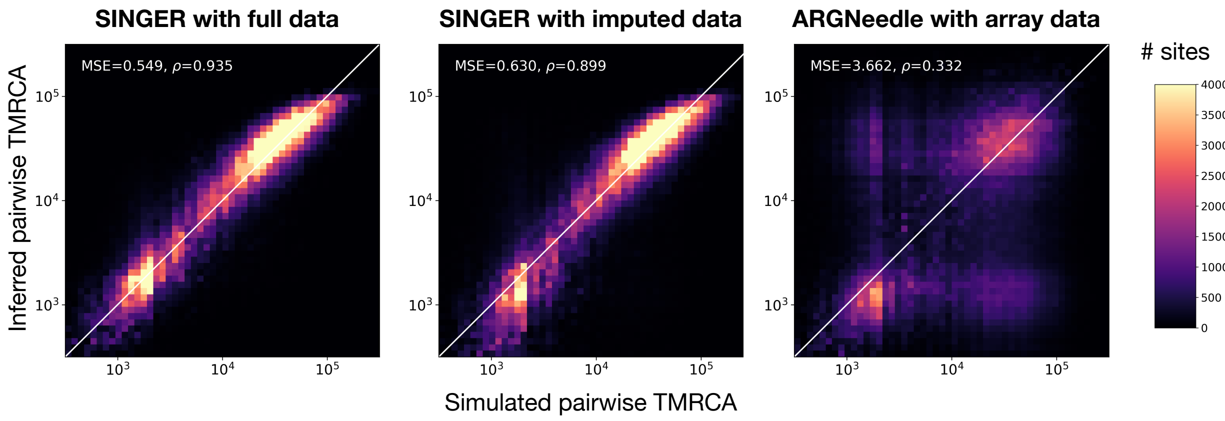

Supplementary Figure 19: Comparison of ARG inference performance for full sequence data using SINGER, imputed sequence data using SINGER, and array data using ARG-Needle.

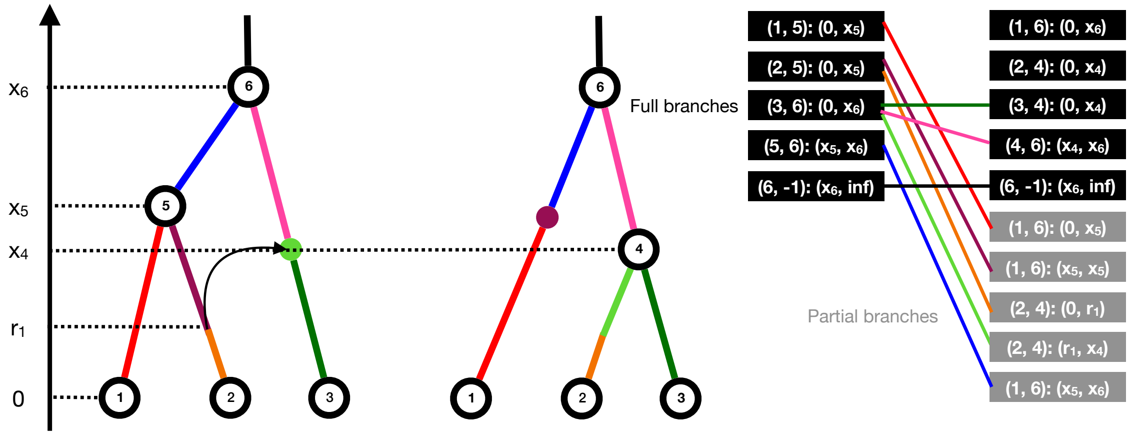

Supplementary Figure 20: Branch correspondence (left) and HMM state spaces (right) before and after a recombination. When there is an existing recombination in the partial ARG, the joining point will shift between segments with the same color. This means if the new node joins the (partial) branch with one color in the previous tree, it must join the (partial) branch with the same color in the next tree, vice versa. On the right, we mark the state transitions with the same color of these segments correspondence. "−1" denotes a point at infinity. Note that a full branch in the previous tree might become a partial branch after the recombination (e.g., the red and blue), a full branch could also become 2 full branches (e.g., pink and dark green) and a branch could also be not affected at all (e.g. black root branch). A state is represented with the branch and the time interval on the branch, and all possible transitions are plotted (right). For example, the red segments correspond to $(1,5):(0,x_5) \rightarrow (1,6):(0,x_5)$ and purple segments correspond to $(2,5):(0,x_5) \rightarrow (1,6):(x_5,x_5)$.

B Method details

The key components of SINGER include branch sampling, time sampling, ARG re-scaling, and Sub-Graph Pruning and Re-grafting (SGPR). Here we describe these procedures in detail. Figure 21 illustrates the terminology we employ.

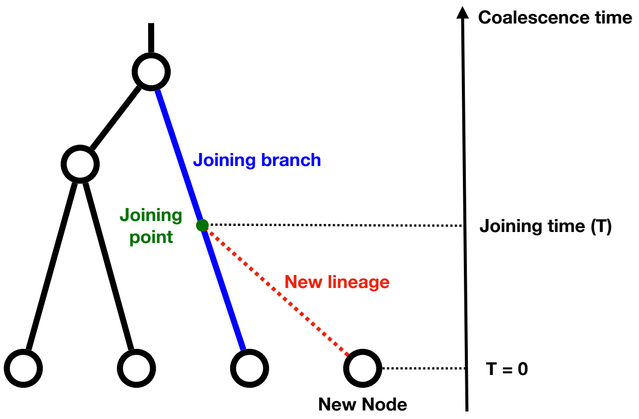

Supplementary Figure 21: Illustration of the terminology used. "New node" refers to the node being threaded onto the partial tree, while "new lineage" refers to the branch connecting the new node to the partial tree. "Joining branch" refers to the branch in the partial tree that the new lineage attaches to, and "joining point" refers to the location of attachment. The time of the joining point is called "joining time".

B.1 Branch sampling

In the branch sampling step, we construct an HMM with branches as hidden states to "thread" a new node onto a sequence of marginal trees. Let S_ℓ denote the state space for bin ℓ. There are two key quantities we need to compute for each branch in the marginal tree: (1) A representative joining time for the branch, which determines the probability of seeing mutations on the new lineage (emission probability in the HMM); and (2) the probability of joining the branch under the coalescent process, which is needed for calculating transition probabilities in the HMM (Section B.1.5). These are detailed below.

B.1.1 Representative joining time and joining probability for branches

For threading a new node onto a single marginal tree Ψ, let T be the joining time of the new node and $\lambda_\Psi(t)$ the number of lineages at time t in the tree. When assuming a pairwise coalescence rate of 1, the exceedance probability $\bar{F}_\Psi(t) := \mathbb{P}_\Psi(T > t)$ and the density function $f_\Psi(t)$ of T are given by

$$\bar{F}_\Psi(t) = \exp\left(-\int_0^t \lambda_\Psi(x)dx\right), \tag{1}$$

$$f_\Psi(t) = -\frac{d}{dt}\bar{F}_\Psi(t) = \lambda_\Psi(t)\bar{F}_\Psi(t). \tag{2}$$

The probability of coalescing onto a branch b_i that spans the time interval $[x, y]$ can be calculated as

$$p_i = \int_x^y \frac{f_\Psi(t)}{\lambda_\Psi(t)} dt = \int_x^y \bar{F}_\Psi(t) dt,$$

where we divide $f_\psi(t)$ by $\lambda_\psi(t)$ because at each time t there are $\lambda_\psi(t)$ possible branches to join and branch b_i is one of them.

We choose a representative joining time τ_i for each branch b_i for the calculation of the transition (Section B.1.5) and emission probabilities (Section B.1.3), as detailed in the next section.

B.1.2 Deterministic approximation

Suppose the partial ARG has n leaf nodes. For $n \gg 1$, $\lambda_\Psi(t)$ is almost deterministic and is well approximated by its expectation (Frost and Volz, 2010)

$$\lambda_\Psi(t) \approx \frac{n}{n + (1-n)\exp(-\frac{t}{2})}.$$

Using this result, we can approximate $\bar{F}_\Psi(t)$, $f_\Psi(t)$, and p_i as

$$\bar{F}_\Psi(t) = \exp\left(-\int_0^t \lambda_\Psi(x)dx\right) \approx \frac{\exp(-t)}{[n + (1-n)\exp(-\frac{t}{2})]^2}$$

$$f_\Psi(t) = \lambda_\Psi(t)\bar{F}_\Psi(t) \approx \frac{n\exp(-t)}{[n + (1-n)\exp(-\frac{t}{2})]^3}$$

$$p_i = \int_x^y \bar{F}_\Psi(t)dt \approx \left\{\frac{-2}{(1-n)^2}\log\left[n + (1-n)\exp(-t/2)\right] - \frac{2n}{(1-n)^2}\frac{1}{n + (1-n)\exp(-t/2)}\right\}\Bigg|_x^y.$$

We choose the representative joining time τ_i for branch b_i using a heuristic:

$$\lambda(\tau_i) = \sqrt{\lambda(x)\lambda(y)}$$
$$\tau_i = \lambda^{-1}(\sqrt{\lambda(x)\lambda(y)}),$$

where

$$\lambda^{-1}(l) = -2\log\left(\frac{n - nl}{l - nl}\right)$$

is the inverse function of $\lambda(\cdot)$. The intuition is that p_i is the difference of a function at $\lambda(x)$ and $\lambda(y)$, and we approximate the midpoint of the function by choosing τ_i so that $\lambda(\tau_i)$ is the geometric mean of $\lambda(x)$ and $\lambda(y)$. This choice works well empirically.

We note that p_i's do not necessarily sum to 1, but they will be used in a "scaled-by-sum" fashion in transition probabilities (Section B.1.5). We apply the above deterministic approximations to all marginal trees in the partial ARG, thereby achieving massive computational savings in branch sampling.

B.1.3 Emission probability

We assume that mutations arise on each branch according to a Poisson point process with intensity $\theta/2$, independently of all other branches. Here, $\theta = 4N_e\mu$, where N_e denotes the reference effective population size and μ the per-bp, per-generation mutation rate. When the new node attaches to the joining branch, it will create a new lineage and bisect the joining branch into two (Figure 22); these

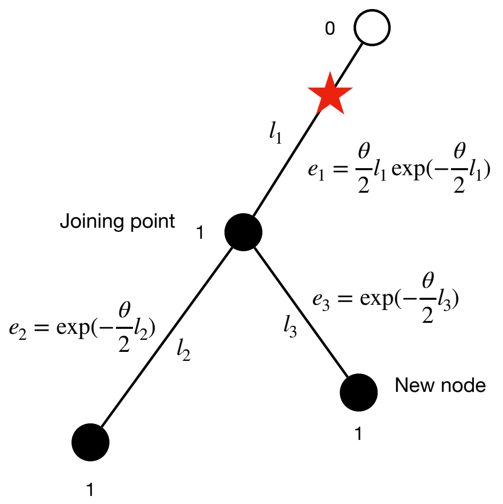

Supplementary Figure 22: The emission probability calculation. We first impute the '0/1' character state at the joining point, which in this case should be 1, so a mutation (red star) has to be placed on the upper half of the joining branch. We can calculate the probability of each of three branches, and the product of them will be the final emission probability.

three branches are involved in the calculation of emission probability. We first impute the '0/1' state at the joining point under parsimony, and calculate the mutation probability for each of the 3 branches. The product of these 3 probabilities is then defined as the per-bp emission probability (Figure 22), and we multiply these probabilities over the base pairs in each bin to obtain the per-bin emission probability.

A special case is when the new lineage joins the branch above the root. Specifically, the allelic state at the new root is set to 0 with probability p_{root} or 1 with probability $1 - p_{\text{root}}$, By default, we use $p_{\text{root}} = 0.5$ for un-polarized data; it can be set to a high value (e.g., 0.99) if the data are polarized, where 0 denotes the ancestral state. We do not make any assumptions on the allelic state of other nodes of the tree. Our recommendations on how to set this parameter can be found at the GitHub documentation: `https://github.com/popgenmethods/SINGER`.

B.1.4 Transitions induced by an existing recombination in the partial ARG

When the partial ARG already contains a recombination event between a given pair of adjacent loci, we forbid the new lineage from introducing any new recombination event between those loci. However, the joining branch for the new lineage can switch between such loci simply by "hitchhiking" with the existing recombing lineage, as detailed below.

If we trace the joining branch in the adjacent marginal trees before and after a recombination, the correspondence is as shown in Figure 20, which is the same as in Rasmussen et al. (2014). Joining a segment with a certain color in the previous tree will necessarily mean joining the segment of the same color in the next tree, and vice versa. For example, if the new node joins the red branch in the first tree, then it has to join the red segment in the second tree; because of the recombination, the upper node of the red branch in the first tree no longer exists in the second tree and the red segment is only a portion of a longer branch in the second tree.

Hence, after a recombination in the partial ARG, some joining branches will be only partial branch segments instead of full branches (e.g., the red and blue segments in the second tree in Figure 20), and these partial segments arise from full branches of a previous tree but extended

to the current position. We refer to these segments as "partial branches". We note that partial branches can go through multiple recombinations along the genome, during which they will become more and more fragmented. The state space for a given site consists of all full branches of the marginal tree at the site and a set of partial branches arising from previous sites. We do not keep track of every partial branch state, but only keep a partial branch state if it has a forward probability (in the forward algorithm of the HMM) larger than a threshold ϵ (usually set at 1%). In other words, we prune unlikely partial branches while running the forward algorithm at the same time. This controls the state-space size to be at most $2n-1+\frac{1}{\epsilon}$. Empirically, we observed that the state space size is only slightly larger than $2n-1$.

When a branch becomes multiple segments (e.g., in Figure 20, the full branch state $(2,5):(0,x_5)$ from the left tree breaks up into $(1,6):(x_5,x_5)$ and $(2,4):(0,r_1)$ in the right tree), the transition probability from the branch will be distributed to the segments proportional to the coalescence probability (obtained by integrating $\frac{f_\Psi(t)}{\lambda_\Psi(t)}$ over their respective time intervals in the branch). A more subtle case in Figure 20 is the full branch state $(3,6):(0,x_6)$ in the left tree which breaks up into three segments. Joining anywhere on the light green segment of the right tree will look like joining the light green dot on the left, so we set the time interval for the light green dot to be the time interval of the recombination arrow.

Why not just use full branch states? Here we explain why using only full branch states can be problematic. Consider the partial ARG shown in Figure 23A, which has three marginal trees spanning one bin each. Suppose a third sequence is being threaded onto it. If we constructed an HMM using only full branches, the state space for each bin would be as shown in Figure 23B. Since the partial ARG already contains recombination events between adjacent loci, the new lineage cannot introduce additional recombinations. In threading the third sequence, the transition $(1,5)\to(3,-1)$ is possible if the new lineage joins the branch $(1,5)$ above the recombination time r_1 in the first bin (Figure 23D). The transition $(3,-1)\to(1,4)$ is also possible if the new lineage joins the branch $(1,5)$ below r_1 in the first bin (Figure 23D). However, the joint move $(1,5)\to(3,-1)\to(1,4)$ is impossible in an ARG without introducing an additional recombination in the new lineage. This is effectively a consequence of decoupling the sampling of topologies and the sampling of coalescence/recombination times. Even under standard Markovian approximations to the ARG generation process, the Markovian property only holds exactly when recombination and coalescence times are represented in the state space. However, we can address this issue by introducing partial branch states.

Our proposed HMM is shown in Figure 23C. Every full branch in a marginal tree must become a hidden state, and the tricky part is birth and death of partial branch states. When transitioning from the first to the second bin, if the state for the first bin is $(1,5):(0,x_5)$, which is a full branch state, it becomes the partial branch state $(1,3):(0,r_1)$ or $(3,-1):(x_5,x_5)$, depending on whether the joining point is lower or higher than the recombination time, respectively. We note that not all partial branch states are kept, as they must have forward probabilities larger than a threshold. Those below the threshold will be dropped, indicated by the red states in Figure 23C. In this example, both blue and green paths are valid in the HMM (Figure 23C), and they correspond to the two different threading operations shown in Figure 23D.

B.1.5 Transitions with a new recombination

Consider a pair of adjacent loci $\ell-1$ and ℓ, and let b_i be the joining branch for bin $\ell-1$ with representative time τ_i. If the partial ARG does not already contain a recombination event between

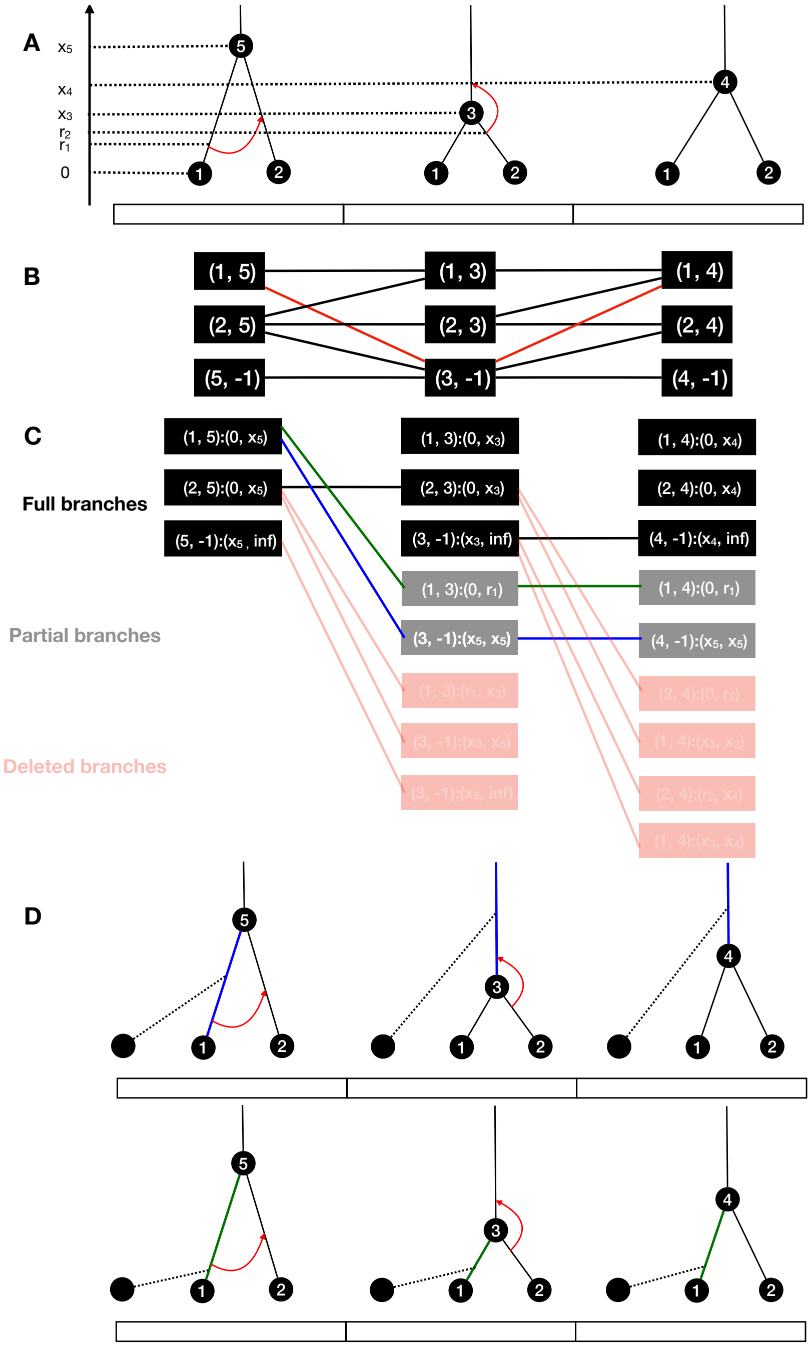

Supplementary Figure 23: An example of the state space in the HMM and why only using full branches as hidden states will not work. Each hidden state consists of a branch and a time interval, and depending on whether the time interval covers the entire branch, a state can be either a full or partial branch, colored black or grey, respectively. The solid lines connecting states between adjacent columns indicate state transitions when no new recombination from the threaded node happens. In this example, because we do not allow more than one recombination to happen between adjacent loci, there should be no more new recombinations. (A) The partial ARG when threading the third leaf node; (B) What the HMM looks like when only using the full branches as hidden states, and the red path is allowed by the HMM but disallowed in an ARG; (C) The HMM with both full and partial branch states. Two valid path are marked with blue and green respectively; (D) The two different valid threadings which correspond to the HMM path in (C).

these adjacent loci, then the new lineage can undergo recombination with probability

$$r_i = 1 - \exp\Big(-\frac{\rho}{2}\tau_i\Big),$$

where $\rho = 4N_e rm$, with r being the per-bp, per-generation recombination probability and m the bin size. The transition probability of the HMM can be defined as

$$\mathbb{P}_\rho(B_\ell = b_j | B_{\ell-1} = b_i) = (1 - r_i)\delta_{ij} + r_i \frac{q_j}{\sum_{k:b_k \in S_t} q_k},$$

for $b_i \in S_{\ell-1}$ and $b_j \in S_\ell$, where

$$q_j = \begin{cases} r_j p_j, & \text{if state } b_j \text{ is a full branch,} \\ 0, & \text{if state } b_j \text{ is a partial branch.} \end{cases}$$

This has the interpretation that with probability $1-r_i$ the new lineage will stay on the same joining branch due to the absence of recombination, whereas if a recombination happens to the new lineage, it will join branch b_j proportional to q_j. The q_j terms for partial branches are always 0, because after a recombination, the new lineage always joins a full branch at bin ℓ. This also means that a partial branch state can be entered only when a full joining branch state from an earlier bin results in a partial branch state due to a recombination in the partial ARG.

In the sequentially Markov coalescent (SMC), given that a recombination happens in the new lineage, the conditional transition probability from b_i to b_j will depend on both b_i and b_j, but to reduce computational complexity we drop the dependence on the previous joining branch and assume that the re-joining probability distribution is the same regardless of the previous joining branch. However, the choice of the re-joining probability distribution still needs to guarantee that the stationary distribution is $\mathbb{P}(B_\ell = b_i) = p_i$, in the most simple case when there is only one marginal tree in the ARG. This leads to choosing the re-joining probability distribution to be $\{q_j / \sum_{k:b_k \in S_\ell} q_k\}$. This also reflects the fact that lower branches are down-weighted when considering the re-coalescence after a recombination, because re-coalescence needs to be more ancient than the recombination event, which forbids joining branches lower than the recombination breakpoint.

B.2 Time sampling

Conditioned on a sequence of joining branches along the genome obtained from branch sampling, the joining times on these branches can be sampled using a modified PSMC (Li and Durbin, 2011) model with the state space for each bin restricted to the time interval of the sampled branch.

We note that although the state space in the branch sampling HMM involve both partial and full branches, only the sequence of full joining branches are used in time sampling step. Partial branches are only used when calculating the transition probability in certain cases (detailed in Section B.2.3 for type B)

This model, which is also an HMM, is detailed below.

B.2.1 Transition and emission probabilities

First, consider the simple case where the joining branch spans the entire non-negative half-line $[0, \infty)$. In this case, conditioned on there being a recombination in the new lineage, the probability density of the joining time transitioning from s to t is given by

$$q_0(t|s) = \int_0^{s\wedge t} \frac{1}{s} e^{-(t-u)} du = \begin{cases} \frac{1}{s}[1 - e^{-t}], & t < s, \\ \frac{1}{s}[e^{-(t-s)} - e^{-t}], & t \geq s. \end{cases}$$

Similarly, if we do not condition on there being a recombination,

$$q_\rho(t|s) = \begin{cases} \frac{1-e^{-\rho s}}{s}[1-e^{-t}], & t < s, \\ e^{-\rho s}, & t = s, \\ \frac{1-e^{-\rho s}}{s}[e^{-(t-s)} - e^{-t}], & t \geq s, \end{cases}$$

and the corresponding cumulative distribution function is

$$Q_\rho(t|s) = \int_0^t q_\rho(x|s)dx = \begin{cases} \frac{1-e^{-\rho s}}{s}[t + e^{-t} - 1], & t < s, \\ \frac{1-e^{-\rho s}}{s}[s - e^{-(t-s)} + e^{-t}] + e^{-\rho s}, & t \geq s. \end{cases} \tag{3}$$

Now, suppose the joining branch at bin ℓ spans the time interval $[x_\ell, y_\ell)$. We partition this time interval into d sub-intervals $[t_{\ell,0}, t_{\ell,1}), [t_{\ell,1}, t_{\ell,2}) \ldots, [t_{\ell,d-1}, t_{\ell,d})$, where $t_{\ell,0} = x_\ell$ and $t_{\ell,d} = y_\ell$, uniformly according to the exponential distribution with rate 1 (the default is partitioning every 5% quantile). In the time sampling HMM, these sub-intervals correspond to the states for bin ℓ. For each sub-interval $[t_{\ell,i}, t_{\ell,i+1})$, we define the representative time $\tau_{\ell,i}$ as

$$\exp(-\tau_{\ell,i}) = \frac{\exp(-t_{\ell,i}) + \exp(-t_{\ell,i+1})}{2}.$$

Then, we define the transition probability from a sub-interval $[t_{\ell-1,i}, t_{\ell-1,i+1}) \subset [x_{\ell-1}, y_{\ell-1})$ at bin $\ell - 1$ to $[t_{\ell,j}, t_{\ell,j+1}) \subset [x_\ell, y_\ell)$ at bin ℓ as

$$q_{i,j}^{\ell-1,\ell} = \frac{Q_\rho(t_{\ell,j+1}|\tau_{\ell-1,i}) - Q_\rho(t_{\ell,j}|\tau_{\ell-1,i})}{Q_\rho(y_\ell|\tau_{\ell-1,i}) - Q_\rho(x_\ell|\tau_{\ell-1,i})}. \tag{4}$$

We define the emission probability in the same way as in branch sampling, depicted in Figure 22.

The state spaces for two consecutive loci in the time sampling HMM can be different if the joining branch changes between those loci. More precisely, there are three cases to be considered, as illustrated in Figure 24: (A) Neither the joining branch nor the partial ARG changes. (B) The partial ARG changes and the joining branch might change (recombination hitchhiking). (C) The partial ARG does not change but the joining branch does (new recombination). We note that both type B and type C transitions will involve either a previous recombination in the partial ARG or a new recombination in threading, but as the number of loci is typically much larger than that of recombinations, the majority of transitions will be of type A.

B.2.2 Linearization of the forward algorithm for type A transitions

The computational complexity for the general HMM forward algorithms is quadratic in the state space size, but, in the case of type A transitions, symmetry structures of the transition matrix in our HMM allow us to reduce the runtime to linear complexity (Harris et al., 2014). We note, however, that time sampling is not the computational bottleneck of SINGER. Because we sample from the posterior using the HMM with stochastic traceback, we only need to implement the forward algorithm.

Here, we follow the arguments in Palamara et al. (2018). For type A transitions between loci $\ell - 1$ and ℓ, note that the state space is the same for the two loci; specifically, $t_{\ell-1,i} = t_{\ell,i}$ for all $i = 0, \ldots, d$. Hence, for ease of notation, we drop the dependence on $\ell - 1$ and ℓ in what follows.

First, note that (3) and (4) imply

$$q_{i,j} = q_{j+1,j}, \quad \text{for all } i > j, \tag{5}$$

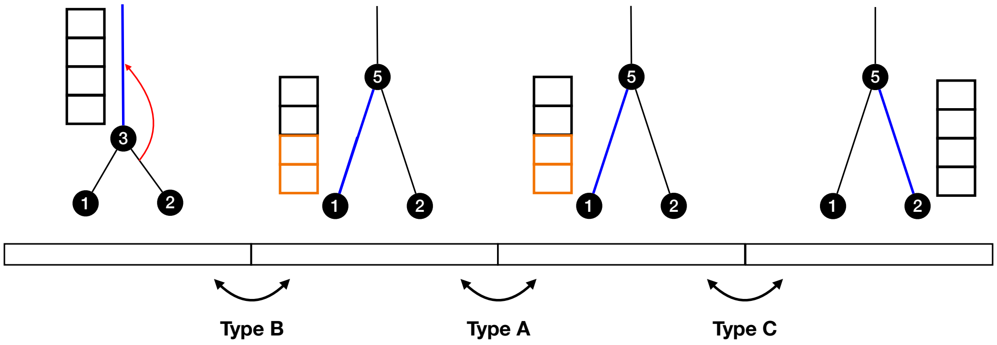

Supplementary Figure 24: An illustration of the state space in the time sampling step. The blue branches denote a sequence of joining branches in different bins. The transition from the first to the second bin corresponds to type B, where the joining branch changes with partial ARG changing as well (recombination hitchhiking). The orange states are created to fill up the branch which is not reached by "recombination hitchhiking"; the transition from the second to the third bin corresponds to type A, where neither the joining branch nor the partial ARG changes. The state space will not change with transitions of type A; the transition from the third to fourth bin corresponds to type C, where the joining branches changes without partial ARG changing (new recombination).

whereas

$$\kappa_j := \frac{q_{i,j}}{q_{i,j-1}} = \frac{\exp(-t_j) - \exp(-t_{j+1})}{\exp(-t_{j-1}) - \exp(-t_j)}, \quad \text{for all } i < j. \tag{6}$$

(The right hand side of (6) does not depend on i, and we call it κ_j.) Let $\alpha_i(\ell)$ denote the forward probability at bin ℓ and state i, which corresponds to the joint probability of observed pairwise data for the first ℓ loci and the hidden state at bin ℓ being i. Let $e_j(\ell+1)$ denote the emission probability at bin $\ell+1$ from hidden state j. Then, the forward algorithm can be written as follows:

$$\begin{aligned}
\alpha_j(\ell+1) &= \sum_i \alpha_i(\ell) q_{i,j} e_j(\ell+1) \\
&= e_j(\ell+1)\left[\sum_{i<j} \alpha_i(\ell) q_{i,j} + \alpha_j(\ell) q_{j,j} + \sum_{i>j} \alpha_i(\ell) q_{i,j}\right] \\
&= e_j(\ell+1)\left[\sum_{i<j} \alpha_i(\ell) q_{i,j} + \alpha_j(\ell) q_{j,j} + \sum_{i>j} \alpha_i(\ell) q_{j+1,j}\right] \\
&= e_j(\ell+1)[S_j + \alpha_j(\ell) q_{j,j} + A_j q_{j+1,j}],
\end{aligned}$$

where $S_j := \sum_{i<j} \alpha_i(\ell) q_{i,j}$, $A_j := \sum_{i>j} \alpha_i(\ell)$, and the third equality follows from (5). Note that S_j and A_j for all $j = 0, \ldots, d-1$ can be computed recursively in linear time using

$$\begin{aligned}
S_j &= \alpha_{j-1}(\ell) q_{j-1,j} + \kappa_j S_{j-1}, \\
A_j &= \alpha_{j+1}(\ell) + A_{j+1},
\end{aligned}$$

with boundary conditions $S_0 = 0$ and $A_{d-1} = 0$, where the recursion for S_j follows from (6).

B.2.3 Type B and type C transitions

Type B transitions. Here, the background partial ARG changes, whereas there may or may not be a change in the joining branch (e.g., the first transition in Figure 24). If the joining branch does not change, then the joining time also does not change in the time sampling HMM.

As we allow at most one recombination between any pair of adjacent loci, the joining branch can change in type B transitions only by "hitchhiking" an existing recombination in the partial ARG as described in Section B.1.4, in a way consistent with the choice of joining branches from the branch sampling process. In this case, each time sub-interval for the joining branch before the recombination is treated as a state in the time sampling HMM, and its corresponding state after the recombination will be constructed only if it is on the sampled joining branch. For example, in Figure 24, the first transition is type B, and the upper two time sub-intervals of the joining branch for the first bin will be on the branch $(5, -1)$ at the second bin after the transition, which is not consistent with the joining branch $(1, 5)$ sampled from the branch sampling process. Hence, these upper two time sub-intervals will not contribute to the calculation of new forward probabilities. Transition probabilities to the upper two sub-intervals on $(1, 5)$ at the second bin are defined in the same way as in Section B.1.4 for partial branches. Finally, when these "hitchhiked" states from before the recombination do not cover the entire branch, we introduce more sub-intervals to fill in the remainder, in the same fashion as in Section B.2.1. In Figure 24, transition probabilities from the first bin to these newly filled states (in orange) at the second bin are defined to be 0, but transition probabilities from the second bin to the orange states at the third bin are non-zero.

Type C transitions. If the joining branch changes between loci ℓ and $\ell+1$ due to a recombination in the new lineage (the third transition in Figure 24), we first build a new state space for bin $\ell + 1$, consisting of time sub-intervals of the new joining branch as described in Section B.2.1. Let $[t_{\ell,0}, t_{\ell,1}), \ldots, [t_{\ell,d_\ell-1}, t_{\ell,d_\ell})$ and $[t_{\ell+1,0}, t_{\ell+1,1}), \ldots, [t_{\ell+1,d_{\ell+1}-1}, t_{\ell+1,d_{\ell+1}})$ respectively denote the state spaces at loci ℓ and $\ell + 1$. Here, since we are already conditioning on having a new recombination (from the branch sampling process), the transition probability can be calculated by setting $\rho = \infty$ in (4). We update the forward probabilities using the standard recursion, as it is not a computational bottleneck in the time sampling HMM:

$$\alpha_j(\ell + 1) = \sum_i \alpha_i(\ell) q_{i,j} e_j(\ell + 1).$$

B.2.4 Inference of recombination times

Although the threading algorithm infers recombination events, it does not infer the exact timing of the recombination breakpoint on the recombining branch.

The recombination time is when the new lineage before and after the recombination get decoupled. The new lineage after recombination will wait from recombination time x until joining time v to re-coalesce with the new joining branch, which we call "re-coalescence event".

To sample the time of the recombination breakpoint, we note that under the SMC, given that the recombination breakpoint occurs at time x, the probability density of the re-coalescence event being at time v is

$$p(x) = e^{-(v-x)}, \quad (7)$$

for $l < x < u$, where l denotes the lower node age of the recombining branch, and u the minimum of the joining times before and after the recombination. For the recombination time, we choose the median according to (7), conditioned on $l < x < u$.

B.3 ARG rescaling

B.3.1 ARG rescaling with constant mutation rate

Given an ARG, we partition the time axis into J intervals $[t_0 = 0, t_1), [t_1, t_2), \ldots, [t_{J-1}, t_J = t_{max})$, where t_{max} is the maximum node age before rescaling, such that in each interval $[t_i, t_{i+1})$ the ARG length is $\frac{1}{J}$ of total ARG length (the default value of J is 100 in our implementation). Here, by "ARG length in an interval", we mean the sum of total branch length overlapping the interval from all marginal trees, weighted by their tree span. Let m_i denote the number of mutations in the interval $[t_{i-1}, t_i)$. We note that some mutations may be mapped to a branch which spans more than one interval, in which case we assign fractions to each interval proportional to the overlap length.

If the total ARG length is $L(G)$, then the expected number of mutations in each time interval should be $\frac{\theta L(G)}{2J}$, so the scaling factor for the interval $[t_{i-1}, t_i)$ needed to match the observation with the expectation should be

$$c_i = \frac{2Jm_i}{\theta L(G)}.$$

We recursively scale and shift the intervals so that $[t_{i-1}, t_i)$ maps to $[\tilde{t}_{i-1}, \tilde{t}_i)$, where $\tilde{t}_0 := 0$ and

$$\tilde{t}_i = c_i(t_i - t_{i-1}) + \tilde{t}_{i-1},$$

for $i = 1, \ldots, J-1$. Then, a coalescence time $t \in [t_{i-1}, t_i)$ is rescaled to

$$\tilde{t} = c_i(t - t_{i-1}) + \tilde{t}_{i-1}.$$

Extended Data Figure 6 illustrates this procedure for the case of a single coalescent tree.

This procedure is similar in spirit to the ARG normalization step in Zhang et al. (2023), as they also post-process the node ages in the ARG to improve time estimates. However, their ARG normalization assumes a known demography to generate the node age distribution by simulation and performs quantile matching based on that information. In contrast, our ARG rescaling strategy is self-contained and only utilizes information from the inferred ages of mutations.

B.3.2 ARG rescaling with mutation rate variation

ARG rescaling can be similar defined with the presence of local mutation rate heterogeneity. We define the local mutation rate function $\mu(x)$ as the local mutation rate as position x, and call the mean mutation rate μ. We can still use the same way to define time windows as B.3.1, but the expected number of mutation will not be $\frac{\theta L(G)}{2J}$. If we denote the branches in the ARG as b_i, their span as $\{(x_i, y_i)\}$ and their lower and upper node ages (l_i, u_i). Then the expected number of mutations in the time interval $[t_i, t_{i+1})$ can be calculated as:

$$\sum_{k=1}^{n} \mu_i \mathbb{I}(x_k < t_{i+1}, y_k > t_i)[\min(y_k, t_{i+1}) - \max(x_k, t_i)]$$

where:

$$\mu_i = \int_{x_i}^{y_i} \mu(s) ds$$

After calculating the expected branch length in each time window given the mutation rate variation, the rescaling can be done in the same way as in B.3.1.

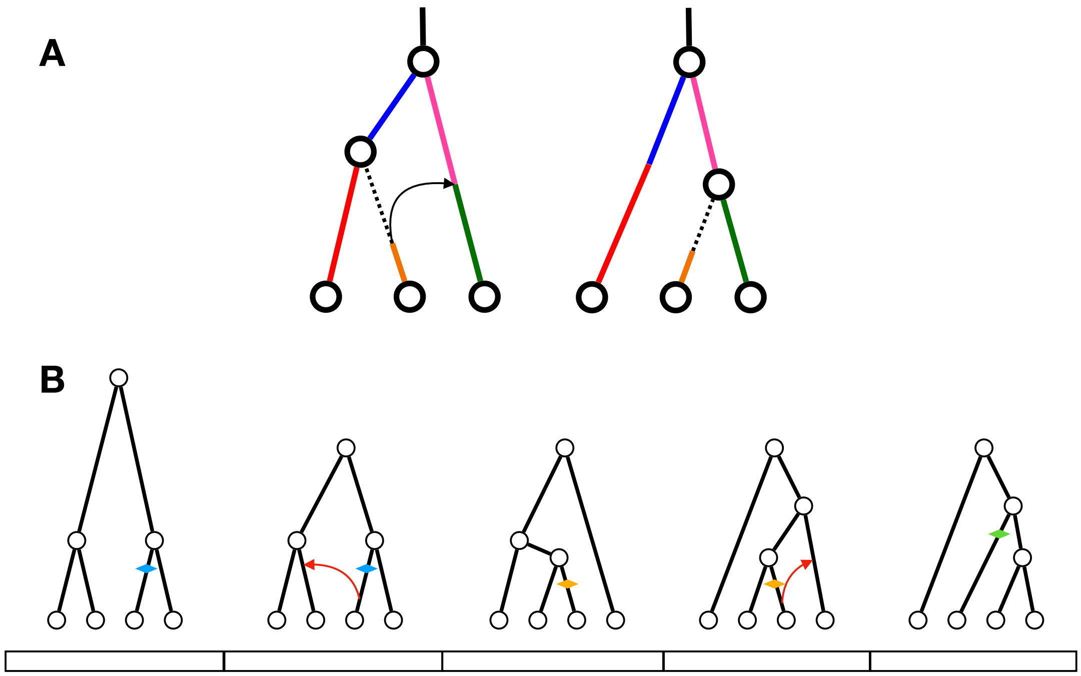

Supplementary Figure 25: The correspondence of cut in adjacent trees and an example of cut extension. (A) If a cut is on the segment with a particular color in the first tree, then the cut will be on the segment with the same color in the second tree (at the same time), vice versa. If the cut is on the dashed segment, then it cannot be extended leftwards or rightwards. (B) In this example, we start by introducing a blue cut to the marginal tree in the first bin, and it extends to and terminates at the second bin. After SGPR induced by the blue cut, a new orange cut is introduced in th marginal tree of third bin, which spans over the third and fourth bin. A green cut in the fifth bin follows the SGPR induced by the orange cut.

B.4 Sub-Graph Pruning and Regrafting (SGPR)

SGPR can be viewed as an extension of the Subtree Pruning and Regrafting (SPR) operation. SPR is a commonly used technique for modifying trees to explore the tree space in phylogenetics, by cutting a subtree and reconnecting it to another location in the remaining tree. In a similar vein, SGPR prunes a sub-graph from an ARG and reconnects it to another part of the remaining graph.

B.4.1 How to prune a sub-graph from an ARG

To prune a sub-graph from an ARG, we first introduce a random cut in a certain marginal tree. The cut usually extends to other trees in the flanking regions. To find the spatial span of the cut, we look for the equivalent point extending leftward and rightward in the tree sequence, where the rule for equivalency is illustrated in Figure 25. A cut located on a colored segment should always correspond to the segment with the same color in the other tree, except when the cut reaches a black dashed line, at which point the extension terminates. For a pair of adjacent loci with a recombination event occurring between them, the black dashed line (Figure 25) in each marginal tree corresponds to the part of the new lineage above the recombination breakpoint; these partial branches get decoupled by the recombination and equivalence of points cannot be established between them (leftwards or

rightwards), and hence the extension of the cut has to terminate. For each marginal tree in the span of the cut, we remove between the cut and the upper node of the branch containing the cut (Extended Data Figure 7).

A cut may or may not span the entire chromosome, and we keep a record of the rightmost position of the span to choose the next cut. If the rightmost position is the end of the chromosome, we start over from the beginning. Otherwise, the next cut will be chosen at the marginal tree at the rightmost position of the previous cut, according to the following steps:

1. Sample a cut time t uniformly at random between 0 and the tree height (tree TMRCA).
2. Determine the branches intercepting time t.
3. Choose one of them uniformly at random and cut the chosen branch at time t.

B.4.2 Comparison of SGPR and the Kuhner move

Here we point out the similarities and differences between the Kuhner move (Kuhner et al., 2000) and our proposals (SGPR). The Kuhner move works with the temporal formulation of the ARG, and starts by picking a branch in the graph (e.g., the green branch in Extended Data Figure 7A). Then, all history (meaning that all coalescence events and recombination events) for the ancestral material corresponding to this branch get removed from the graph (Extended Data Figure 7B). Since we can translate the temporal representation of the ARG into a spatial representation comprising a sequence of marginal trees, the ancestry removal operation in the Kuhner move leads to a sequence of trees with portions of branches removed (Extended Data Figure 7B). In other words, one can think of it as introducing a cut to a marginal tree and propagating the cut leftwards and rightwards. For every marginal tree affected, the part of the branch above the cut is removed (Extended Data Figure 7B). This removal operation of the Kuhner move is the same as in SGPR.

The main difference is in the regraft step (Extended Data Figure 7), in that the Kuhner move does this by simulating the coalescent with recombination process from the prior distribution, starting from the cut. This is intuitively problematic because the regrafted genealogy receives no information from the data and might not be consistent with the data. This problem can be seen from the acceptance ratio of the Kuhner move (Mahmoudi et al., 2022):

$$A(G \to G') = \min\left\{1, \frac{B(G)\mathbb{P}(G'|D)}{B(G')\mathbb{P}(G|D)}\right\},$$

where $B(G)$ is the number of branches in the graph and $\mathbb{P}(G|D)$ is the likelihood of graph G given the data D. This result implies that unless the likelihood of the newly proposed graph G' is substantially better than before, the proposal will be unlikely to be accepted. However, this is very hard to achieve if the prior distribution is employed to complete the ARG after the removal step. As suggested in Mahmoudi et al. (2022), although the Kuhner move seems necessary and sufficient for good mixing when $\mu = 0$, it results in poor convergence for real data with mutations.

In our proposal (SGPR), we instead use threading to sample from the approximate posterior. This proposal now takes the data into account and is more likely to reach good likelihoods. In Section B.4.3, we show that when assuming that the threading algorithm approximately samples from posterior, the acceptance ratio will not contain the likelihood ratio term, which means we can introduce big moves in the proposal while having good acceptance probability.

We also point out that the removal scheme of SGPR and the Kuhner move can be seen as a clarification of the branch selection algorithm in ARGweaver (Figure 26). ARGweaver removes a sequence of branches by using a data structure called the “branch graph”, and given the removed

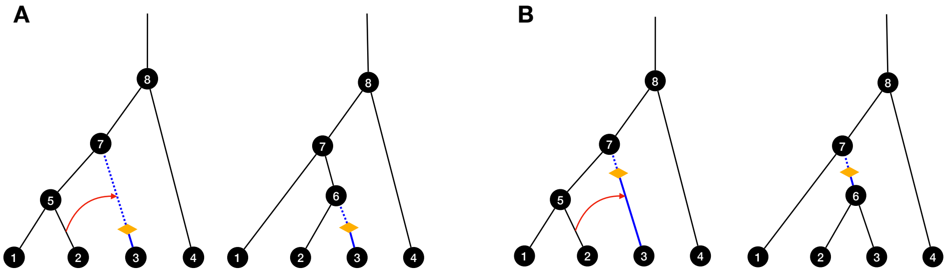

Supplementary Figure 26: Similarities and differences between SGPR and ARGweaver proposals. When ARGweaver chooses branch $(3, 7)$ to remove in the first tree, there are two possible branches to remove in the second tree, namely either $(3, 6)$ or $(6, 7)$. The two cases can be induced by having different locations of the cut on the same branch $(3, 7)$ in the first tree (a lower cut in A versus a higher cut in B). Orange oval: a cut in the SGPR move; blue branches: affected branches; blue dashed parts: removed partial branches.

branch in the current tree, there can be up to two choices for removing a branch in the next tree. We illustrate with an example in Figure 26 that if we specify a location of the cut in the previous branch, then the next branch choice is always unique. If we do not know the location of the cut, then there can indeed be two possible next branches to choose from.

B.4.3 How to regraft the sub-graph to generate an updated ARG

After pruning a sub-graph, we need to regraft it to generate an updated ARG. In our proposal, we use our threading algorithm to re-thread the cut point onto the remaining graph H after pruning the sub-graph, to obtain a new ARG state G'. This leads to a substantial improvement over the Kuhner move because the threading algorithm considers the data and will find good genealogies consistent with the observed allelic patterns. We show that with the following acceptance ratio, the MCMC will achieve detailed balance:

$$A_H(G \to G') = \min \left\{ 1, \frac{\mathbb{P}(G'|D) q_H(G' \to G)}{\mathbb{P}(G|D) q_H(G \to G')} \right\},$$

where $q_H(G \to G')$ denotes the probability of updating G to G' via the sub-graph pruning from G to H and re-grafting from H to G'.

Proof. The target distribution of the MCMC is $\mathbb{P}(G|D)$ and we have

$$\begin{aligned} &\mathbb{P}(G|D) \int_H q_H(G \to G') A_H(G \to G') dH \\ &= \int_H \min \left\{ \mathbb{P}(G|D) q_H(G \to G'), \mathbb{P}(G'|D) q_H(G' \to G) \right\} dH \\ &= \mathbb{P}(G'|D) \int_H q_H(G' \to G) A_H(G' \to G) dH. \end{aligned}$$

Hence, detailed balance is achieved with the stationary distribution being $\mathbb{P}(G|D)$. □

Now, suppose the threading algorithm in the regraft step can approximately sample from the

posterior distribution. Then,

$$\begin{aligned} q_H(G \to G') &= \mathbb{P}(H|G)\mathbb{P}(G'|D, H) \\ &= \mathbb{P}(H|G)\frac{\mathbb{P}(G'|D, H)}{\int_{G'':H\in\mathcal{H}(G'')}\mathbb{P}(G''|D, H)} \\ &= \mathbb{P}(H|G)\frac{\mathbb{P}(G', H|D)}{\int_{G'':H\in\mathcal{H}(G'')}\mathbb{P}(G'', H|D)} \\ &= \mathbb{P}(H|G)\frac{\mathbb{P}(G'|D)}{\int_{G'':H\in\mathcal{H}(G'')}\mathbb{P}(G''|D)}, \end{aligned}$$

where $\mathcal{H}(G'')$ denotes the set of all subgraphs of G'', and $G'' : H \in \mathcal{H}(G'')$ means the set of all ARGs that can reach H after a sub-graph pruning, which is the same as the set of all ARGs that H can reach after regrafting. The last equality follows since H is a sub-graph of both G' and G''. Using this proposal distribution, the acceptance ratio can then be calculated as

$$A_H(G \to G') = \min\left\{1, \frac{\mathbb{P}(H|G')}{\mathbb{P}(H|G)}\right\}.$$

Now, for the proposal scheme described in Section B.4.1, we have

$$\mathbb{P}(H|G) = \frac{1}{h(\Psi_x)},$$

where $h(\Psi_x)$ is the height of the tree Ψ_x at the rightmost position x of the previous cut.

The acceptance ratio now reduces to a very simple form, involving a ratio between the tree height before and after the proposal:

$$A_H(G \to G') = \min\left\{1, \frac{h(\Psi_x)}{h(\Psi'_x)}\right\}.$$

Consistent with intuition, we later show empirically that this ratio of tree heights is typically very close to 1 for sufficiently large sample sizes, meaning that few rejections will occur while making big updates to the ARG.

B.4.4 Metropolis-Hastings acceptance rate comparison between SINGER and ARGweaver

We also compared the Metropolis-Hastings acceptance rates of ARGweaver and SINGER, for 50 sequences in the same computational experiments as mentioned in Online Methods of the main text. We note that there are two types of MCMC proposals in ARGweaver – namely, leaf-rethreading and subtree-rethreading – each with 50% probability. Leaf-rethreading is a Gibbs sampling procedure that has an acceptance rate of 1; however, it is inefficient in exploring the ARG space because the conditional coalescence is most likely to happen in the lower time ranges and hence unlikely to perturb the deep structures of the ARG. As SGPR corresponds to the concept of subtree-rethreading in ARGweaver, we directly compared the acceptance rates of SGPR and subtree-rethreading. ARGweaver only reports the average acceptance rate across all chunks of genome segment (100 kb by default), so we examined that statistic in our comparison. On average, ARGweaver accepts the subtree-rethreading proposal move at a rate of only $\sim 22\%$, while SGPR moves in SINGER are accepted with a rate close to 1 Figure 1.

B.4.5 Runtime of SINGER

The runtime of threading in SINGER is linear in the sequence length times the number of haplotypes in the partial tree, so initializing iterative threading for all sequences has a computational complexity of $\mathcal{O}(nL)$, where n is the number of haplotypes and L the sequence length. As each MCMC iteration performs re-threading on the full ARG, it has a complexity of $\mathcal{O}(nL)$, as all haplotypes are already in the ARG. The total computational complexity of SINGER is thus $\mathcal{O}(mnL + n^2L)$, where m denotes the number of MCMC iterations. We note that $\mathcal{O}(nL)$ is only the worst- case complexity for re-threading in MCMC, as only branches higher than the cut point are allowed to be re-grafted onto. In practice, many re-threading in MCMC has a much smaller state space than the leaf threading, as the cut points can often be high in coalescent trees.

C Simulation benchmarks

C.1 Comparison with PSMC in inferring pairwise TMRCA

Since many ARG-based applications involve inferring pairwise coalescence times (Fan et al., 2022; Zhang et al., 2023; Wang and Coop, 2022), it is of interest to know whether using multi-sequence ARG inference methods leads to more accurate estimates than separately carrying out pairwise inference using PSMC models, especially extremely fast versions like XSMC (Ki and Terhorst, 2020) and Gamma-SMC (Schweiger and Durbin, 2023). Here, we investigate this question for Gamma-SMC with 50 sequences (the same setting as the constant population size scenario in Online Methods of the main text).

Surprisingly, despite modeling the joint genealogy of the entire sample, which should contain more information than just a pair of sequences, comparing Figure 2 with Figure 2A suggests that SINGER can substantially outperform Gamma-SMC, whereas Relate and tsinfer+tsdate do not seem to improve on Gamma-SMC in terms of MSE or correlation.

C.2 Unscaled total variation distance

To compare the ground truth and inferred genealogies more globally rather than examining low-dimensional summary statistics, we used the total variation distance introduced in Zhang et al. (2023), with a slight modification.

Here, we recap the idea of the total variation distance. In a given tree, each branch defines a descendant clade and the clade can be represented as a binary string in $\{0,1\}^n$, where n is the number of leaf nodes. A '1' at position i in the binary string means that the i-th leaf node is in the clade and otherwise not. A function $f : \{0,1\}^n \to \mathbb{R}$ can be constructed by defining $f(x)$ to be the branch length if x is the binary string corresponding to a branch in the tree and $f(x) = 0$ otherwise. We can then define a distance between two trees by using the L_1 norm of the difference between their corresponding functions. An example is provided in Figure 4.

In Zhang et al. (2023), the aforementioned functions are normalized to sum to 1 before calculating the L_1 distance, and it is referred to as the "total variation distance". In our setting, to account for the inference accuracy of the total tree length, we did not perform this normalization. Also, for methods that used MCMC to explore the tree topology, we used the mean function over the posterior samples to calculate the total variation distance.

We used the unscaled total variation distance to benchmark the inferred ARGs against simulated ARGs for 50 sequences under the constant population size model and the CEU demography model. SINGER was able to achieve the smallest distance for both scenarios (Figure 5).

C.3 Robustness to model misspecification

To assess the robustness to model misspecification, especially that of population size history, we performed simulations with $N_e = 10,000$ but inferred ARGs assuming $N_e = 2,000$ (Figure 6). Further, we performed simulations under an inferred population size history for CEU (estimated using SMC++ (Terhorst et al., 2017) and available at `https://github.com/PalamaraLab/ASMC_data/tree/main/demographies` and applied ARGs methods assuming a constant $N_e = 10,000$ (Figure 2B). For both scenarios, SINGER outperformed the other methods in terms of the accuracy of estimated pairwise coalescence times.

C.4 The impact of thinning on rank plots

Thinning interval can be an important parameter in MCMC samplers, as samples get less correlated with larger thinning intervals. In the rank plot analysis, one may argue that the U-shape is due to a lack of thinning. Here, we tried using 10 times larger thinning intervals (200 iterations) for ARGweaver than SINGER, and the suggested saturated thinning (1000 iterations) for Relate (Extended Data Figure 3A).

All samplers deviated less from the uniform distribution with more thinning, but ARGweaver still performed worse in pairwise TMRCA, rank plot, and CI coverage compared to SINGER (Extended Data Figure 3B), even with 10 times more iterations (which means hundreds to thousands times longer runtime!). Relate, however, seems to have reached saturated thinning, because the rank plots change little when thinning interval length is increased from 1000 to 1500. Still, the rank plots are not flat (Extended Data Figure 3A). We believe this has to do with the fact that Relate does not sample tree topologies.

C.5 Expected site frequency spectrum

Ralph et al. (2020) pointed out the general duality between the statistics of the genome and the genealogy. In particular, the expected site frequency spectrum (SFS) can be calculated from the ARG by integrating over all possible mutation histories. It is important that the expected SFS from inferred genealogies matches the observed SFS from genetic variation data, and deviation from it indicates flaws in the algorithm design.

We compared the expected SFS from inferred ARGs with the SFS from simulated data, under both a constant population size demography and the CEU demography model (Figure 7). For the constant size model, the results of ARGweaver and SINGER are similar, and they deviate much less from the simulated SFS compared to Relate and tsinfer+tsdate. For the CEU model, the expected SFS for SINGER still matches the observed SFS well, but ARGweaver displays a bigger deviation.

C.6 Recombination rate heterogeneity

It is well known that there is substantial recombination rate variation in the human genome (Myers et al., 2005). To carry out more realistic simulation benchmarks, we simulated 50 sequences using fine-scale recombination maps inferred by pyrho (Spence and Song, 2019), under a constant size model with other settings the same as in Online Methods. To be more precise, we picked the recombination map for a 10-20 Mb region from chromosome 1 in GBR (Figure 8).

We then compared SINGER's performance when using the fine-scale recombination map versus when using only the average recombination rate (Figure 9). As expected, inferred ARGs are more accurate when the fine-scale recombination map is used, but using the average recombination rate still works well. This suggests that if high-quality recombination maps are available, they may be used to increase the inference accuracy.

D Applications to the 1000 Genomes Project

D.1 Data and parameters for running ARG inference methods

We used 200 whole genomes from 5 African indigenous populations (GWD, YRI, ESN, LWK, and MSL) in the 1000 Genomes Project, with 40 genomes drawn uniformly at random from each population. The per-generation per-bp mutation rate (μ) and recombination rate (r) were both set to 1.2×10^{-8} in SINGER. In order to determine the effective population size N_e, we matched the empirical average pairwise difference ($\pi \approx 0.001$) with the theoretical expectation ($4N_e\mu$), which led to a choice of $N_e = 20,000$. We ran SINGER for 10,000 iterations, with the first 4,000 iterations as burn-in. We then took 100 samples from the rest, thinning every 60 iterations.

To carry out some of the empirical benchmarks, we additionally ran SINGER and Relate on the whole genome sequences of GBR in the 1000 Genomes Project (Byrska-Bishop et al., 2022). Based on the same reasoning as above, we chose $\mu = 1.2 \times 10^{-8}$ and $N_e \approx 15,000$. As Relate needs the data to be polarized, we downloaded the ancestral human genome from `https://personal.broadinstitute.org/konradk/loftee_data/GRCh38/`. For tsinfer+tsdate, we extracted the GBR genealogies from the ARG inferred by Wohns et al. (2022), as it includes all individuals from the 1000 Genomes Project.

D.2 Storage efficiency and the runtime of computing relevant statistics from inferred ARGs

We note that different ARG representations lead to different storage efficiencies. For example, in terms of the output format, the most notable difference between Relate and tsinfer is that the latter requires explicit node/edge sharing across adjacent marginal trees, while the former does not enforce this in terms of storage and it might store equivalent branches multiple times. Similar to tsinfer, SINGER also requires explicit node/edge sharing and leverages the succinct tree sequence data structure of tskit (Kelleher et al., 2016).

This data structure choice has a direct impact on storage efficiency since the explicit use of node/edge sharing can make the output file much more compact (Table 1). As a comparison, SINGER takes 2.4 GB space for a single sample of genome-wide ARG, similar to tsinfer+tsdate (2.1 GB), whereas Relate takes 17 GB. Notably, SINGER inferred one order of magnitude more recombination breakpoints than Relate and tsinfer (Table 1) without requiring a much larger output file size, resulting in more information and a better compression ratio. Given that the recombination and mutation rates are similar in humans, and the total number of segregating sites is about 1.19×10^7, the number of marginal trees inferred by SINGER seems more consistent with the data.

The benefit of having explicit node/edge sharing is not simply restricted to storage efficiency. For computing genome-wide statistics from an inferred ARG, often the marginal tree sequences have to be scanned. If most nodes/edges are shared between adjacent trees, the update operations will be of low computational cost. For example, to compute the genome-wide average pairwise TMRCAs over 1 kb windows from an inferred ARG, it takes Relate 197 s, tsinfer 5 s and SINGER 18 s (Table 1). We note that SINGER can output multiple ARG samples, which would increase storage and computational cost, but it would still be feasible to carry out genome-wide analyses.

	SINGER	tsinfer+tsdate	Relate
File Size	2.4 GB	2.1 GB	17 GB
Number of Trees	1.23×10^7	1.18×10^6	1.06×10^6
Computation time of 1 kb average pairwise TMRCA	18 s	5 s	197 s

Supplementary Table 1: Comparison of storage efficiency and the runtime of computing summary statistics of ARGs inferred by different methods.

D.3 Large-scale diversity patterns

It has long been known that diversity levels are not constant along the genome, even on a rather large scale, e.g., 1 Mb (McVicker et al., 2009). Specifically, if we partition the genome into 1 Mb windows and compute their respective diversities, they will show up to 5-fold differences; this pattern is difficult to explain by a neutral model where demographic perturbations, in average, affects all genomic regions in the same way. A popular hypothesis proposed to explain this phenomenon is that background selection in the form of purifying selection acting on functional sites and affecting other sites through linkage, shapes the genome-wide diversity (McVicker et al., 2009; Murphy et al., 2022).

From the ARG inferred by Wohns et al. (2022) using tsinfer+tsdate, we extracted the sub-ARG for the individuals in GBR and compared it to the ARGs inferred by SINGER and Relate. In particular, for SINGER and tsinfer+tsdate, we calculated branch-length-based diversity estimates for each 1 Mb window, defined as the product of the mutation rate and the average pairwise distance in the ARG for the window, and compared them with the SNP-based diversity estimate from the VCF file. We observed that tsinfer+tsdate severely underestimated genome-wide diversity levels and the extent of their variation, while SINGER yielded much closer fits (Extended Data Figure 4A). This suggests a better potential for analyzing background selection, and genome-wide patterns on variability in general, with SINGER.

For SINGER, it is interesting to note that the initial ARG sample does not fit the large-scale diversity pattern as well in some regions, but the fit gets substantially better with MCMC updates (Figure 10A). Besides accounting for the uncertainty in coalescence times and topology, this result demonstrates the benefit of performing MCMC rather than simply using the initial sample. We suggest inspecting such diversity plots as a part of the strategy for monitoring the convergence of the MCMC algorithm implemented in SINGER.

D.4 Convergence diagnostic of SINGER

To examine the convergence of the MCMC chain in SINGER, we looked at the fit of branch-length-based diversity predictions in inferred ARGs to SNP-based diversity in 1 Mb windows for real data.

The deviation (measured as the mean squared error) decreased until a stable level was reached, indicating convergence (Figure 10A).

Additionally, we inspected the mutational mappings. Due to statistical phasing errors, gene conversions, sequencing errors, method imperfections etc, there will be some mutations that are not uniquely mappable to a single branch of a marginal tree, but the number should decrease with MCMC iterations (Figure 10B). We used a burn-in of 4,000 samples, after which the stabilization of both statistics suggested that the chain reached stationarity.

D.5 Fine-scale diversity estimation

In this section, we focus on the accuracy of estimating fine-scale diversity from ARG inference methods. By "fine-scale diversity", we mean average pairwise coalescence times (scaled by $4N_e\mu$) for small genomic windows. There are two ways of estimating this quantity: (1) by applying the classical nucleotide diversity estimator to the sequence data; or (2) by first inferring the underlying ARG and then calculating the average pairwise coalescence time in the ARG (scaled by $4N_e\mu$). We call them "SNP-based diversity" and "branch-length-based diversity", respectively.

In the main text, we use fine-scale diversity to search for regions with population-specific reductions in diversity. Strong local reductions in genetic diversity are often used to identify sites that warrant further evolutionary analysis. To accurately attribute the signal to a gene or regulatory element, genetic diversities need to be estimated at fine scales, e.g., 1 kb resolution. However, using SNP-based diversity can be very noisy at fine scales (Figure 11A), thereby limiting its utility. In general, for SNP-based diversity, there is a trade-off when choosing window sizes for diversity measures in which too small a window size causes high variance along the length of the genome, and too large window sizes causes over-smoothing where the signal may be lost. However, if the ARG was directly observable, it would provide a much more precise measure of the reduction in diversity/coalescence times. We illustrate this in Figure 11, where the ground truth from simulations is the branch-length-based diversity at each specific genomic coordinate. If posterior ARG samples from SINGER are used to obtain posterior averages of branch-length-based diversities, the agreement with the ground truth can be substantially improved (Figure 11D,E) compared to SNP-based estimates. In contrast, this benefit of using branch-length-based diversity estimates from inferred ARG is not as pronounced for Relate (Figure 11B), and even worse than SNP-based estimates for tsinfer+tsdate (Figure 11C).

D.6 Previously reported selection targets in Britain

We analyzed the candidate loci for positive selection in Britain identified in Mathieson and Terhorst (2022) and characterized them in two different ways using the inferred ARGs. First, we compared the average pairwise TMRCA in 1kb windows between GWD and GBR samples across the genome (Figure 12). The candidate loci fall toward the right end of the distribution, indicating substantially deeper coalescence times in these loci in Africans than in British individuals. This makes sense because they have been under strong positive selection in British but not necessarily so in Africans. We note that *LCT* is not a particularly strong outlier, probably because of the convergent evolution of lactase persistence in both Europeans and Africans (Tishkoff et al., 2007). Second, we adopted the visualization approach from Nielsen et al. (2025) to compare the average pairwise TMRCA among the carriers of a beneficial allele to that of all samples combined (Extended Data Figure 5). We chose the candidate target SNP of selection for each gene based on previous literature (Enattah et al., 2002; Wang et al., 2010; Canela-Xandri et al., 2018; Salvo et al., 2023) and found signals consistent with selective sweeps. These signals are characterized by contiguous segments of reduced

pairwise TMRCA among the carriers of the target SNP compared to the overall samples.

D.7 Introgression analysis

For the introgression analysis described in the main text, we inferred the site-specific distribution of pairwise coalescence times between a given leaf and all other leaves. However, the marginal distribution for a single tree is rather degenerate because it consists of a collection of point masses with different multiplicities (Figure 16A). In contrast, combining the trees sampled from the posterior using SINGER, which typically have different branch lengths and topologies, can substantially smooth this distribution (Figure 16B).

We aimed to show that ARG-based methods can distinguish introgression tracts reported by reference-based methods. To this end, we checked whether SINGER and Relate show distinct features in the introgression tracts reported by IBDmix, which works by directly comparing the genomes of modern humans and Neanderthals. We chose introgression regions longer than 100 kb in the entire genome and created a set of control windows of the same size. Based on this, we created a balanced sample of test and control regions of 100 kb long, and calculated relevant statistics for Relate and SINGER genealogies. We note that IBDmix only reports individual-level introgression tracts, so we took the larger ratio from the two haplotypes as the putative introgressed region.

For Relate, we calculated the proportion of long branches for each leaf node, as suggested by Speidel et al. (2019). For each window, the proportion of long branches is defined as the genome span in which the ancestral lineages of a leaf node contain a long branch that spans over the introgression window, divided by the window length. For both Relate and SINGER, we calculated the coalescence ratio as mentioned in the main text (see the section on Archaic introgression). The introgression window was chosen to be 60 kya–500 kya (Skoglund and Mathieson, 2018). Figure 6B shows the ROC curve and the AUROC value for SINGER and Relate (Figure 6B). SINGER slightly outperforms Relate, regardless of whether coalescence ratio or long branch proportion is used.

We note that the upper right part of Relate's ROC curve is straight. The reason for this behavior is that there are regions without any long branches spanning over the introgression window at all, even in the reported introgression tracts. So, when the threshold changes, the true positive rate and the false positive rate will suddenly increase to include all these windows without any long branches, which comprise a non-trivial portion of the reported introgression tracts (as well as control windows). This is in line with the fact that long branches are not robust to incorrect topology inference (Figure 15) and that even in reported introgression tracts, the proportion of long branches can still be low, sometimes even zero.

D.8 Other loci with ancient coalescence time

We also computed genome-wide fine-scale diversity in every 1 kb window to search for loci with exceptionally old coalescence times, using SINGER's inferred ARG and the efficient implementation of "diversity(mode='branch')" API in tskit (Kelleher et al., 2018). We note that, in order to avoid mappability issues, we also applied the Umap filter (Karimzadeh et al., 2018) and only included the loci with mappability scores $> 95\%$ to improve the reliability of the results. We also used the mutation rates inferred by Roulette (Seplyarskiy et al., 2023) to remove hyper-mutated regions (50 kb windows with the average mutation rate exceeding the genome-wide 95th percentile). We note that balancing selection does not necessarily lead to ancient coalescence times or even trans-species polymorphism, which depends on the timescale and the strength of balancing selection. Exceptionally old coalescence time can arise from long-term balancing selection, but can be consistent with other forces, too. Here, we made a Manhattan plot of the genome-wide fine-scale diversity estimates (Figure 18) and highlighted a few loci with exceptionally old coalescence times (above the 99.99th percentile of the non-HLA region), some of which have been previously reported to be under long-term balancing selection, such as *ABO*, *TRIM5*, *IGFBP7*, *PKD1L1*, *DMBT1* (Ségurel et al., 2012; Leffler et al., 2013; Bitarello et al., 2023; Cagliani et al., 2010). The full list can be found at `https://zenodo.org/records/14232714`.

E Additional Supplementary Information

E.1 Array data

To assess what would be the best practice when it comes to array data, we carried out 10 replicates of coalescent simulations each with 800 sequences of size 1 Mb, under the CEU demography model as in https://github.com/PalamaraLab/ASMC_data/tree/main/demographies, with $\mu = 1.65 \times 10^{-8}$ and $r = 1.2 \times 10^{-8}$. We use the first 300 sequences as the test dataset and the remaining 500 as the reference panel. To mimic realistic array data, for the test data we additionally performed rejection sampling of the SNPs based on their frequency so that the adjusted site frequency spectrum matches the empirical spectrum in the UK Biobank. We note that this is very similar to the simulation setting of Zhang et al. (2023), as described in https://zenodo.org/records/7745746.

We compared two ways of performing ARG inference for array data: first, we directly ran ARG-Needle on the simulated array data; second, we first used Beagle 5.4 (Browning et al., 2018) to impute the test array data using the remaining 500 sequences as a reference panel and then ran SINGER on the imputed data. We additionally ran SINGER directly on the original test data (before rejection sampling) to assess the extent of accuracy loss from using imputed data. For pairwise TMRCA inference, we observed that applying SINGER to imputed sequence data is much more accurate than applying ARG-Needle directly to array data (Figure 19). Furthermore, compared to using the original full sequence data, the reduction in accuracy from using imputed data is small. Hence, our recommendation for handling array data is to first perform imputation with a good reference panel and then run ARG inference methods.

References

Bitarello, B.D., Brandt, D.Y., Meyer, D., Andrés, A.M., 2023. Inferring balancing selection from genome-scale data. Genome biology and evolution 15, evad032.

Browning, B.L., Zhou, Y., Browning, S.R., 2018. A one-penny imputed genome from next-generation reference panels. The American Journal of Human Genetics 103, 338–348.

Byrska-Bishop, M., Evani, U.S., Zhao, X., Basile, A.O., Abel, H.J., Regier, A.A., Corvelo, A., Clarke, W.E., Musunuri, R., Nagulapalli, K., et al., 2022. High-coverage whole-genome sequencing of the expanded 1000 genomes project cohort including 602 trios. Cell 185, 3426–3440.

Cagliani, R., Fumagalli, M., Biasin, M., Piacentini, L., Riva, S., Pozzoli, U., Bonaglia, M., Bresolin, N., Clerici, M., Sironi, M., 2010. Long-term balancing selection maintains trans-specific polymorphisms in the human trim5 gene. Human Genetics 128, 577–588.

Canela-Xandri, O., Rawlik, K., Tenesa, A., 2018. An atlas of genetic associations in uk biobank. Nature genetics 50, 1593–1599.

Enattah, N.S., Sahi, T., Savilahti, E., Terwilliger, J.D., Peltonen, L., Järvelä, I., 2002. Identification of a variant associated with adult-type hypolactasia. Nature genetics 30, 233–237.

Fan, C., Mancuso, N., Chiang, C.W., 2022. A genealogical estimate of genetic relationships. The American Journal of Human Genetics 109, 812–824.

Frost, S.D., Volz, E.M., 2010. Viral phylodynamics and the search for an 'effective number of infections'. Philosophical Transactions of the Royal Society B: Biological Sciences 365, 1879–1890.

Harris, K., Sheehan, S., Kamm, J.A., Song, Y.S., 2014. Decoding coalescent hidden Markov models in linear time, in: Research in Computational Molecular Biology: 18th Annual International Conference, RECOMB 2014, Pittsburgh, PA, USA, April 2-5, 2014, Proceedings 18, Springer. pp. 100–114.

Karimzadeh, M., Ernst, C., Kundaje, A., Hoffman, M.M., 2018. Umap and bismap: quantifying genome and methylome mappability. Nucleic acids research 46, e120–e120.

Kelleher, J., Etheridge, A.M., McVean, G., 2016. Efficient coalescent simulation and genealogical analysis for large sample sizes. PLoS Computational Biology 12, 1–22. doi:10.1371/journal.pcbi.1004842.

Kelleher, J., Thornton, K.R., Ashander, J., Ralph, P.L., 2018. Efficient pedigree recording for fast population genetics simulation. PLoS Computational Biology 14, e1006581.

Ki, C., Terhorst, J., 2020. Exact decoding of the sequentially markov coalescent. bioRxiv , 2020–09.

Kuhner, M.K., Yamato, J., Felsenstein, J., 2000. Maximum likelihood estimation of recombination rates from population data. Genetics 156, 1393–1401.

Leffler, E.M., Gao, Z., Pfeifer, S., Ségurel, L., Auton, A., Venn, O., Bowden, R., Bontrop, R., Wall, J.D., Sella, G., et al., 2013. Multiple instances of ancient balancing selection shared between humans and chimpanzees. Science 339, 1578–1582.

Li, H., Durbin, R., 2011. Inference of human population history from individual whole-genome sequences. Nature 475, 493–496.

Mahmoudi, A., Koskela, J., Kelleher, J., Chan, Y.b., Balding, D., 2022. Bayesian inference of ancestral recombination graphs. PLOS Computational Biology 18, e1009960.

Mathieson, I., Terhorst, J., 2022. Direct detection of natural selection in bronze age britain. Genome Research 32, 2057–2067.

McVicker, G., Gordon, D., Davis, C., Green, P., 2009. Widespread genomic signatures of natural selection in hominid evolution. PLoS Genetics 5, e1000471.

Murphy, D.A., Elyashiv, E., Amster, G., Sella, G., 2022. Broad-scale variation in human genetic diversity levels is predicted by purifying selection on coding and non-coding elements. Elife 12, e76065.

Myers, S., Bottolo, L., Freeman, C., McVean, G., Donnelly, P., 2005. A fine-scale map of recombination rates and hotspots across the human genome. Science 310, 321–324.

Nielsen, R., Vaughn, A.H., Deng, Y., 2025. Inference and applications of ancestral recombination graphs. Nature Reviews Genetics 26, 47–58.

Palamara, P.F., Terhorst, J., Song, Y.S., Price, A.L., 2018. High-throughput inference of pairwise coalescence times identifies signals of selection and enriched disease heritability. Nature Genetics 50, 1311–1317.

Ralph, P., Thornton, K., Kelleher, J., 2020. Efficiently Summarizing Relationships in Large Samples: A General Duality Between Statistics of Genealogies and Genomes. Genetics 215, 779 LP – 797. URL: http://www.genetics.org/content/215/3/779.abstract, doi:10.1534/genetics.120.303253.

Rasmussen, M.D., Hubisz, M.J., Gronau, I., Siepel, A., 2014. Genome-wide inference of ancestral recombination graphs. PLoS Genetics 10, e1004342.

Salvo, N.M., Andersen, J.D., Janssen, K., Meyer, O.L., Berg, T., Børsting, C., Olsen, G.H., 2023. Association between variants in the oca2-herc2 region and blue eye colour in herc2 rs12913832 aa and ag individuals. Genes 14, 698.

Schweiger, R., Durbin, R., 2023. Ultrafast genome-wide inference of pairwise coalescence times. Genome Research 33, 1–9.

Ségurel, L., Thompson, E.E., Flutre, T., Lovstad, J., Venkat, A., Margulis, S.W., Moyse, J., Ross, S., Gamble, K., Sella, G., et al., 2012. The abo blood group is a trans-species polymorphism in primates. Proceedings of the National Academy of Sciences 109, 18493–18498.

Seplyarskiy, V., Koch, E.M., Lee, D.J., Lichtman, J.S., Luan, H.H., Sunyaev, S.R., 2023. A mutation rate model at the basepair resolution identifies the mutagenic effect of polymerase iii transcription. Nature Genetics 55, 2235–2242.

Skoglund, P., Mathieson, I., 2018. Ancient genomics of modern humans: the first decade. Annual review of genomics and human genetics 19, 381–404.

Speidel, L., Forest, M., Shi, S., Myers, S.R., 2019. A method for genome-wide genealogy estimation for thousands of samples. Nature Genetics 51, 1321–1329.

Spence, J.P., Song, Y.S., 2019. Inference and analysis of population-specific fine-scale recombination maps across 26 diverse human populations. Science Advances 5, eaaw9206.

Terhorst, J., Kamm, J.A., Song, Y.S., 2017. Robust and scalable inference of population history from hundreds of unphased whole genomes. Nature Genetics 49, 303–309.

Tishkoff, S.A., Reed, F.A., Ranciaro, A., Voight, B.F., Babbitt, C.C., Silverman, J.S., Powell, K., Mortensen, H.M., Hirbo, J.B., Osman, M., et al., 2007. Convergent adaptation of human lactase persistence in africa and europe. Nature genetics 39, 31–40.

Wang, S., Coop, G., 2022. A complex evolutionary history of genetic barriers to gene flow in hybridizing warblers. bioRxiv URL: https://doi.org/10.1101/2022.11.14.516535.

Wang, T.J., Zhang, F., Richards, J.B., Kestenbaum, B., Van Meurs, J.B., Berry, D., Kiel, D.P., Streeten, E.A., Ohlsson, C., Koller, D.L., et al., 2010. Common genetic determinants of vitamin d insufficiency: a genome-wide association study. The Lancet 376, 180–188.

Wohns, A.W., Wong, Y., Jeffery, B., Akbari, A., Mallick, S., Pinhasi, R., Patterson, N., Reich, D., Kelleher, J., McVean, G., 2022. A unified genealogy of modern and ancient genomes. Science 375, eabi8264.

Zhang, B.C., Biddanda, A., Gunnarsson, Á.F., Cooper, F., Palamara, P.F., 2023. Biobank-scale inference of ancestral recombination graphs enables genealogical analysis of complex traits. Nature Genetics 55, 1–9.
